# Repurposing thioridazine as a novel treatment for melanoma brain metastasis

**DOI:** 10.1186/s12935-026-04440-x

**Published:** 2026-08-06

**Authors:** Tobias Wikerholmen, Erlend Moen Taule, Katharina Sarnow, Emma Rigg, Hege Avsnes Dale, Terje Sundstrøm, Jian Wang, Frits Thorsen

**Affiliations:** 1 Department of Biomedicine, University of Bergen, Jonas Lies vei 91, Bergen, 5009 Norway; 2 Department of Neurosurgery, Boston Children’s Hospital, 300 Longwood Ave, Boston, MA 02115 USA; 3 Molecular Imaging Center, Department of Biomedicine, University of Bergen, Jonas Lies vei 91, Bergen, 5009 Norway; 4 Department of Clinical Medicine, University of Bergen, Jonas Lies vei 87, Bergen, 5009 Norway; 5 Department of Neurosurgery, Haukeland University Hospital, Haukelandsveien 22, Bergen, 5021 Norway; 6 Department of Neurosurgery, Qilu Hospital of Brain and Brain Inspired Science, Shandong University, Jinan, 250012 China; 7 Jinan Microecological Biomedicine Shandong Laboratory and Shandong Key Laboratory of Brain Function Remodeling, Jinan, 250117 China

**Keywords:** Melanoma, Brain metastases, Thioridazine, Cancer, Metastases, Antipsychotics, Autophagy, Brain tumor, Neuroleptic, Drug-repurposing

## Abstract

**Background:**

A substantial proportion of patients with melanoma brain metastases (MBM), especially those symptomatic at therapy start, continue to respond poorly to currently available systemic therapies, including immune checkpoint inhibitors. Durable systemic treatment options beyond established regimens remain limited for this patient population. Drug repurposing offers a rapid translational path for identifying novel therapeutic strategies. Thioridazine, a phenothiazine antipsychotic with known blood–brain barrier permeability, has shown anticancer effects in several malignancies but has not previously been evaluated in MBM. This study investigates the anti-tumoral potential and underlying mechanisms of thioridazine in patient-derived MBM cell lines.

**Methods:**

Cytotoxic, anti-proliferative, and anti-migratory effects of thioridazine were assessed in four MBM cell lines (H1, H2, H3, H10) using monolayer and soft agar viability assays, live-cell imaging, and clonogenic assays. Potential off-target toxicity was evaluated using fetal rat brain organoids (FRBOs). Mechanistic effects were examined using Annexin V/PI flow cytometry, Western blotting of apoptosis- and autophagy-related proteins (cleaved PARP1, cleaved caspase-3, Bcl-2, p62, LC3A/B), and confocal microscopy to assess autophagosome formation and lysosomal dynamics.

**Results:**

Thioridazine reduced viability of MBM cells in a dose-dependent manner, with IC_50_ values ranging from 8.7 to 12.0 µM. Anchorage-independent growth was similarly inhibited. FRBOs displayed no treatment-associated cytotoxicity at MBM-relevant doses in vitro. Thioridazine markedly suppressed MBM cell proliferation, migration, and clonogenic growth in a dose-dependent manner. Apoptosis induction was inconsistent across cell lines, with variable activation of cleaved PARP1, cleaved caspase-3, and Bcl-2. In contrast, autophagy-associated markers p62 and LC3A/B were consistently upregulated, accompanied by intracellular accumulation of punctate autophagosomes and altered lysosomal morphology, indicating impaired autophagic flux.

**Conclusions:**

Thioridazine demonstrates robust anti-tumoral activity in MBM cells in vitro while sparing healthy brain organoid tissue. Its cytotoxic effects appear independent of classical apoptosis and instead correlate with disrupted autophagic flux and altered lysosomal dynamics. These findings highlight thioridazine as a promising candidate for therapeutic repurposing in MBM and support further investigation into its autophagy-modulating mechanisms.

**Supplementary Information:**

The online version contains supplementary material available at https://doi.org/10.1186/s12935-026-04440-x.

## Background

The incidence of melanoma, a cancer of melanocytes, is steadily increasing in Norway and other Scandinavian countries [1]. Equally concerning is melanoma’s high propensity to metastasize to the brain. Clinical studies report that 30–60% of patients with metastatic melanoma develop melanoma brain metastases (MBM), while autopsy studies reveal MBM in 70–90% of patients with disseminated disease [2]. As a result, the management of MBM is becoming an increasing challenge for both patients and clinicians.

The standard treatment regimen for MBM typically involves a combination of surgery and radiation, followed by a clinically guided approach using immunotherapy and targeted therapies. Immunotherapy has revolutionized the management of metastatic melanoma, showing durable survival in a subset of responding patients [3]. However, treatment response is highly variable and is strongly reduced in patients who require supportive corticosteroids for symptom control. As a result, a substantial proportion of MBM patients do not achieve disease control with immunotherapy [3–5]. Small-molecule inhibitors targeting common oncogenic drivers in MBM, such as BRAF mutations, also play a key role in treatment; however, most patients only experience short-term responses before disease progression occurs [6, 7]. Current efforts to develop novel therapies and optimize existing treatment strategies are advancing rapidly, yet many emerging therapies are still several years away from clinical implementation [8–10]. This has positioned drug repurposing, using approved, well-characterized therapeutics for new indications, as a low-risk yet high-reward strategy for accelerating clinical translation [11].

In addition to well-known challenges in cancer therapy, such as tumor heterogeneity and drug resistance, treating MBM introduces unique obstacles due to the distinct characteristics of the central nervous system (CNS). A key barrier to the effective treatment of CNS metastases is the blood–brain barrier (BBB), a physiological barrier within the brain vasculature composed of tightly connected endothelial cells, supported by astrocytic end-feet, pericytes, and efflux transporters. The BBB thereby restricts the passage of molecules between the bloodstream and the brain parenchyma [12]. However, the protective role of the BBB also limits the entry of almost all therapeutic agents, making drug delivery to the brain particularly challenging. Strategies to bypass the BBB include direct approaches, such as intrathecal injections, as well as biochemical approaches that utilize small, lipophilic drugs capable of crossing the BBB [12]. In this context, antipsychotic drugs are of particular interest, as they are already approved for clinical use, exert their effects within the brain, and possess favorable properties for BBB penetration.

Building on this rationale, our laboratory has previously investigated the effects of antipsychotics on malignant brain tumors [13, 14]. Notably, thioridazine showed encouraging effects in glioblastoma models when combined with temozolomide, prompting further investigation in the context of MBM [14]. Thioridazine is a piperidine phenothiazine antipsychotic that exerts its therapeutic effects primarily through dopamine receptor antagonism. However, the drug is not exclusive to dopamine receptor D2 (DRD2), but also binds to other receptors such as serotonin, noradrenaline, and histamine receptors [15]. Its relatively low molecular weight (370.6 Da) and high lipophilicity make it well suited for CNS penetration, even in the presence of an intact BBB [16].

Thioridazine has previously been investigated both as monotherapy and in combination with established chemotherapeutics across a wide range of cancers, including glioma, glioblastoma, melanoma, osteosarcoma, prostate, colon, lung, breast, ovarian, cervical, endometrial, and renal cancers [14, 17–29]. Thioridazine has also shown direct effects on cancer stem cells, which are hypothesized to contribute to the formation of distant metastases [21, 30–34]. Interestingly, thioridazine seems to inhibit the growth of melanoma cells that have developed resistance to standard treatments [35].

The precise basis underlying the anticancer activity of thioridazine remains poorly understood. Proposed mechanisms for thioridazine (or phenothiazines in general) in cancer include induction of autophagy [14, 21, 23, 26, 33, 36] promotion of apoptosis [19, 27, 29], inhibition of angiogenesis [37], calmodulin inhibition [38], membrane damage [39], cytoskeletal disruption [40], inhibition of DNA repair [24, 25], and modulation of cellular efflux pumps [41]. However, the anticancer effects appear to be independent of binding to its traditional therapeutic target, DRD2 [18, 21].

Given that most studies reporting thioridazine-related apoptosis involve its use in combination with other established apoptosis-inducing chemotherapeutics, it is likely that other mechanisms than apoptosis contribute to thioridazine’s antitumoral effects. In this context, thioridazine has been proposed to interfere with autophagy in cancer cells, potentially explaining the enhanced apoptotic response observed when combined with chemotherapeutic agents due to the accumulation of drugs and cellular waste products. Autophagy can be described as the process by which cells degrade and recycle intracellular components to maintain homeostasis [42]. In cancer, autophagy likely exhibits dual roles, acting as a tumor suppressor in early stages of tumor formation, while later contributing to tumor cell survival and therapeutic resistance [43].

In this work, we investigated the potential of thioridazine as a new therapeutic strategy for MBM and characterized its molecular and cellular effects in patient-derived MBM cell lines in vitro.

## Materials and methods

### Cell lines and cell culture

The Regional Ethical Committee (REC) approved the tissue collection and biobank storage of the tumor biopsies and the development and use of the cell lines (REC Approvals 2013/720 and 2020/65185). Written informed consent was obtained from all patients. Cell lines were authenticated by short tandem repeat (STR) fingerprinting and regularly tested for mycoplasma.

NHA cells were purchased from the American Type Culture Collection (ATCC; Rockville, MA, USA). The H1, H2, H3, and H10 cell lines were established in our laboratory from MBM patient biopsies. Primary cutaneous tumor locations were as follows, H1, dorsal torso; H2, scapula; H3, thigh; H10, ventral torso. Patient biopsies used for culture initiations were obtained during surgical resection. None of the patients had received any systemic treatment prior to surgery. The BRAF mutational status of the H1, H2, H3, and H10 cell lines was determined by massive parallel sequencing of the tumor DNA. The H1, H2, and H10 cell lines are BRAF^V600E^-mutated, while the H3 cell line is BRAF^L577F^-mutated.

All cells were grown in Dulbecco’s modified Eagle´s medium (DMEM; Sigma-Aldrich Inc., St. Louis, MO, USA), supplemented with 10% heat-inactivated newborn calf serum (Thermo Fisher Scientific, Waltham, MA, USA), 5 µg/mL Plasmocin (Invivogen, Toulouse, France), 2% L-glutamine (BioWhittaker, Verviers, Belgium), penicillin (100 IU/mL), and streptomycin (100 µL/mL) (BioWhittaker), hereafter referred to as culture medium. The cells were cultured in a standard tissue incubator at 37 °C, 100% humidity, and 5% CO_2_ and trypsinized at 70–80% confluency, using 0.25% Trypsin/EDTA (Thermo Fisher Scientific, cat. no. 25200056).

During experiments requiring the seeding of a specific number of cells, the cells were cultured, trypsinized, centrifuged, and resuspended in culture medium before being stained with trypan blue (Thermo Fisher Scientific, cat. no. T10282). The cells were then counted using Countess™ Cell Counting Chamber Slides (Thermo Fisher Scientific, cat. no. C10228) on a Countess™ automated cell counter (Invitrogen, Waltham, MA, USA).

### Drug

Thioridazine (cat. #3070/50) was purchased from R&D Systems (Minneapolis, MN, USA) and dissolved in dimethylsulfoxide (DMSO). 50 mM aliquots were stored at -20 °C until further use.

### Monolayer cell viability assays

Monolayer cell viability was assessed using a WST-1 assay (Roche, Basel, Switzerland, cat. #11644807001). Cells were seeded at a density of 5 × 10^3^ cells per well in 96-well plates (Thermo Fisher Scientific, cat. no. 167008) with 100 µL of culture medium per well and allowed to adhere for 24 h. Thioridazine was added at final concentrations of 1, 5, 7.5, 10, 15, 25, or 30 µM and left for 72 h while control wells received culture medium without the drug.

Each experiment included at least three separate wells for each condition. After 72 h of treatment, 100 µL of medium was removed from each well and replaced with 10 µL of WST-1 solution, followed by incubation for 3 h at 37 °C. Absorbance was measured at 620 and 450 nm using a plate reader (Multiscan FC Microplate Photometer, Thermo Fisher Scientific) with SkanIt software (Thermo Fisher Scientific). Values were background-corrected by subtracting the blank from all measurements and IC_50_ doses were calculated using GraphPad Prism version 9 (GraphPad Software, Inc., San Diego, CA, USA). The experiments were done in triplicate.

In parallel, cell morphology following thioridazine treatment was studied using a Nikon TE2000 inverted microscope (Nikon Instruments Inc., Melville, NY, USA).

### Tumorsphere cell viability assay

Thioridazine’s effect on anchorage-independent growth and viability of H1, H2, and H3 cells was then studied. Cells were suspended at 1.6 × 10^5^ cells/mL and mixed 1:1 with 0.6% low-melting-point agarose (Sigma-Aldrich Inc.), yielding a final solution of 8 × 10^4^ cells/mL. A 50 µL aliquot of the agar-cell solution was then seeded onto 50 µL of cooled 0.6% Difco noble agar (BD Biosciences, San Jose, CA, USA) into each well of a 96-well plate (Thermo Fisher Scientific, cat. no. 167008), resulting in a total volume of 100 µL and 4 × 10^3^ cells per well. The low-melting-point agarose layer with cells was overlaid with 100 µL of fresh culture medium, and the plate was incubated for 24 h. Wells were then treated with thioridazine at increasing concentrations (2.5, 5, 10, 15, 20, or 40 µM) or left as untreated controls receiving only fresh culture medium, and incubated for an additional 72 h.

After thioridazine treatment, the medium in all wells was replaced with fresh drug-free culture medium, and the plates were incubated for 21 days, with 100 µL of culture medium replaced every 3 days. At the end of the incubation period, 100 µL of culture medium was removed from the wells, and 10 µL of CCK-8 solution (Dojindo Molecular Technologies Inc., Rockville, MD, USA) was added to each well. The cells were then incubated for 3 h at 37 °C. The absorbance was measured at 450 nm using a plate reader (Thermo Fisher Scientific, Multiscan FC Microplate Photometer) and analyzed with SkanIt software (Thermo Fisher Scientific), representing a viability assay.

Each experiment included three wells (*N* = 3) for each condition, and all experiments were performed in triplicate. Microscopy images were obtained using a Nikon Eclipse Ti2 microscope (Nikon Instruments Inc.). Data analysis was performed using GraphPad Prism version 9 (GraphPad Software, Inc.).

### Generation of Fetal Rat Brain Organoids (FRBOs)

Pregnant Sprague-Dawley rats (Janvier Laboratories, Le Genest-Saint-Isle, France) were sacrificed on the 18th day of gestation, and fetuses were surgically removed. Under sterile conditions, the fetal brain tissue was removed, combined, and homogenized with scalpel blades. Brain tissue was washed twice with PBS (Thermo Fisher Scientific, cat. no. AM9624) and enzymatically dissociated with StemPro Accutase (Thermo Fisher Scientific, cat. no. A1110501) in a 37 °C water bath for 20 min, followed by manual dissociation with a serological pipette. Meninges and other floating debris were removed, and the remaining sedimented tissue was resuspended in culture medium and filtered through a 70-µm cell strainer (Thermo Fisher Scientific, cat. no. 08-771-2).

After two washes in culture medium, single cells were counted manually with a hemocytometer after staining with Trypan Blue. Only live cells were counted and included in the calculations. 2.0 × 10^6^ viable cells were seeded in agar-coated 24-well plates (Thermo Fisher Scientific, cat. no. 142485). After three days, aggregated cells were transferred to agar-coated T75 flasks (Thermo Fisher Scientific, EasYFlask, cat. no. 156499). Culture medium changes were performed every 72 h for the first 14 days and every 48 h from day 14 to day 21.

### Live/Dead Viability Assay for FRBOs

A Live/Dead assay was performed to assess the viability of FRBOs after thioridazine treatment. 21-day-old FRBOs were transferred to low-attachment 96-well plates and treated with 10 µM thioridazine for 48 h. Untreated organoids served as controls. FRBOs were washed and stained with the LIVE/DEAD™ Viability/Cytotoxicity Kit for mammalian cells (Thermo Fisher Scientific, cat. no. L3224) according to the manufacturer’s protocol. Briefly, FRBOs were incubated for 40 min at 37 °C with 2 µM calcein AM and 4 µM ethidium homodimer-1, transferred to 8-well µ-slides with a glass bottom (Ibidi, Gräfelfing, Germany, cat. no. 80826), and imaged using an Andor Dragonfly 505 confocal microscope (Andor Technology, Belfast, Northern Ireland).

### Proliferation assay

The proliferative capability of MBM cells was studied under continuous exposure to thioridazine for 72 h, using a live-cell imaging system. H1, H2, H3, and H10 cell lines were seeded at a density of 5 × 10^3^ cells/well in Essen BioScience ImageLock 96-well plates (Essen BioScience Ltd., Hertfordshire, UK, cat. no. 4379) in 100 µL of prewarmed culture medium. After 24 h, 100 µL of culture medium containing thioridazine at 2 x experimental concentrations was added to the wells, resulting in final concentrations of 2.5, 5, 10, or 20 µM thioridazine (*n* = 6 wells per treatment group). Control wells received prewarmed culture medium only.

The culture plate was placed in an IncuCyte^®^ Live Cell Imaging System (Essen BioScience Ltd.) and imaged every 2 h using a 10 x objective. The experiment was performed in triplicate (*n* = 3 per experiment per drug concentration). The images were masked and analyzed for confluency using the built-in IncuCyte^®^ Base Analysis Software. The raw output from this analysis was further explored and graphically presented using GraphPad Prism version 9 (GraphPad Software, Inc.).

### Cell migration assay

The migratory capacity of MBM cells treated with thioridazine was assessed through a scratch wound assay. H1, H2, H3, and H10 cells were seeded in ImageLock 96-well plates (Essen BioScience Ltd., cat. no. 4379) and incubated for 48 h at 37 °C. Seeding densities were as follows: NHA − 3 × 10^4^, H1–3 × 10^4^, H2–2.5 × 10^4^, H3–2.5 × 10^4^, H10–2.5 × 10^4^ cells. After 48 h, a wound-making tool (Essen BioScience Ltd., IncuCyte wound-maker tool) was used to create a consistent and uniform wound across all wells.

50 µL of the cell culture medium was removed from each well and replaced with 50 µL of pre-warmed fresh culture medium to remove floating cells. The wells then received 100 µL of fresh cell culture medium containing thioridazine at 2 x concentrations (5, 10, 20, or 40 µM), resulting in final well concentrations of 2.5, 5, 10, or 20 µM. Control wells only received pre-warmed culture medium. Each drug concentration was tested in at least three replicate wells per experiment.

The plates were then placed in an IncuCyte^®^ Live-Cell Imaging System (Essen BioScience Ltd.) and imaged every 2 h for 72 h using a 10 × objective. Wound closure was analyzed using the IncuCyte^®^ Scratch Wound Cell Migration Software Module (Essen BioScience Ltd., cat. no. 9600-0012). Raw data from the built-in IncuCyte^®^ software were further analyzed in GraphPad Prism version 9 (GraphPad Software, Inc.) for final figure production. The experiment was performed in triplicate (*N* = 3 independent experiments per drug concentration).

### Clonogenic assay

Clonogenic assays were performed to assess the potential loss of reproductive integrity of MBM cells. NHA, H1, H2, H3, and H10 cell lines were seeded at a density of 200 cells/well in 2 mL culture medium in 6-well plates (Thermo Fisher Scientific, cat. no. 140675) and allowed to adhere for 24 h. The cells were then treated with thioridazine at concentrations of 2.5, 5, or 10 µM for 72 h. Untreated cells served as controls. After treatment, the cells were washed with PBS (Thermo Fisher Scientific, cat. no. AM9624), replenished with fresh culture medium, and incubated for an additional 12–14 days. After this incubation period, the culture medium was removed and the cell layer was washed with cold PBS (Thermo Fisher Scientific, cat. no. AM9624). The cells were then fixed with pre-chilled methanol at − 20 °C for 10 min. The methanol was removed, and the plates were left to dry at room temperature. The cell colonies were then stained using 0.5% crystal violet in 25% methanol in water for 10 min, washed three times with distilled water, and dried.

The plates were imaged using a Nikon TE2000 inverted microscope (Nikon Instruments Inc.), and colonies containing more than 50 cells were counted using ImageJ/Fiji (National Institutes of Health, Bethesda, MD, USA). Representative images were obtained to verify colony formation. Data analysis and graph preparation were carried out using GraphPad Prism version 9 (GraphPad Software, Inc.). Statistical analysis was performed using a two-way ANOVA with multiple comparisons, and *p* < 0.05 was considered statistically significant. The experiment was performed in triplicate (*N* = 3 independent experiments per drug concentration).

### Apoptosis assay by flow cytometry

Apoptosis was assessed using the AlexaFluor^®^ 488 Annexin V/PI apoptosis kit (Thermo Fisher Scientific, cat. no. V13245). H1, H2, H3, and H10 cells were seeded at 2 × 10^5^ cells in 5 mL of growth medium in T25 flasks (Thermo Fisher Scientific, EasYFlask, cat. no. 156367). After 24 h, thioridazine was added to the flasks at final concentrations of 2.5, 5, 10, and 20 µM. Untreated control cells received fresh medium. All cells were then incubated for an additional 72 h.

After 72 h, the culture medium from each flask treated with different thioridazine concentrations was collected into separate Falcon tubes (Sarstedt, Nümbrecht, Germany, cat. no. 62.554.502). The cell monolayers were washed with PBS, and the wash solution was added to the corresponding tubes. Remaining adherent cells were trypsinized using 0.25% Trypsin/EDTA (Thermo Fisher Scientific, cat. no. 25200056), collected, and combined with the respective tubes. Samples were centrifuged at 900 rpm for 4 min, and the supernatants were discarded.

Cell pellets containing both dead and viable cells were resuspended in 100 µL of Annexin binding buffer (provided in the kit) and transferred to 1.5 mL Eppendorf tubes. Cells were stained with 4 µL Annexin V and 1 µL propidium iodide (Thermo Fisher Scientific, cat. no. V13245) and incubated on ice in the dark for 15 min prior to analysis.

The cell suspensions were analyzed using a BD Accuri C6 flow cytometer (BD Biosciences, San Jose, CA, USA). Fluorescence in the FITC-A and PE-A channels was gated using a two-parameter histogram and analyzed with FloJo software (Tree Star Inc., Ashland, OR, USA). The experiment was performed in triplicate (*N* = 3 independent experiments per drug concentration). Data analysis was carried out using GraphPad Prism version 9 (GraphPad Software, Inc.). Statistical analysis was performed using ordinary one-way ANOVA with multiple comparisons. A p-value of less than 0.05 was considered statistically significant.

### Western blot analysis

H1, H2, H3, and H10 cells were treated with thioridazine at a concentration of 7.5 µM for 72 h. Control cells were kept in fresh culture medium for the same duration. The cells were lysed using an ice-cold radioimmunoprecipitation assay (RIPA) lysis buffer (Thermo Fisher Scientific, cat. no. 09901) supplemented with a cocktail of protease inhibitors (Roche, cat. no. 4693124001) and phosphatase inhibitors (Roche, cat. no. 04906837001). The cell lysates were centrifuged at 12,000 rpm for 5 min at 4 °C, and the supernatants were collected for Western blot analysis.

Protein concentration was determined using a Pierce™ BCA Protein Assay (Thermo Fisher Scientific, cat. no. 23225). Western blot gels (NuPAGE™ Bis-Tris Mini Protein Gels, 4–12%, 1.5 mm, Thermo Fisher Scientific, cat. no. NP0336) were loaded with 20 µg of total protein of each cell lysate and resolved by SDS-PAGE. The separated proteins were transferred to PVDF membranes (Sigma-Aldrich, St. Louis, MO, USA, cat. no. IPVH10100), which were subsequently blocked in Tris-buffered saline (TBS) containing 0.1% Tween and 5% skim milk (blocking buffer) at room temperature (RT). After washing twice in 0.1% Tween containing TBS (TBS-T), the membranes were incubated overnight at 4 °C in blocking buffer containing the following primary antibodies: rabbit-p62 (1:1000; Cell Signaling, Danvers, MA, USA, cat. no. 8025 S), rabbit LC3A/B (1:1000; Cell Signaling, cat. no. 12741 S), rabbit cleaved PARP1 (1:1000; Abcam, Cambridge, UK, cat. no. ab32561), rabbit cleaved caspase-3 (1:1000; Cell Signaling, cat. no. 9664 S), rabbit Bcl-2 (1:1000; Abcam, cat. no. 32124). Loading controls used were GAPDH (1:5000; Abcam, cat. no. 9485) and β-actin (1:4000; Abcam, cat. no. ab8224).

Membranes were then washed five times in TBS-Tween and incubated for 1 h with a 1:5000 dilution of a goat anti-rabbit IgG (H + L) cross-adsorbed HRP-conjugated secondary antibody (Beckman Coulter, cat. no. IM0831) in blocking buffer. After washing the membranes five times, the proteins were detected using an enhanced chemiluminescence kit (Thermo Fisher Scientific, cat. no. A43841 or cat. no. 37074) and imaged using a LAS3000 imaging system (FujiFilm, Tokyo, Japan). The protein expression levels were quantified based on the density of the protein bands detected using ImageJ/Fiji software version 1.54p (National Institutes of Health). The relative protein levels were first normalized to the loading control and then calculated and presented as ratios relative to untreated controls. The experiments were performed in triplicate (*N* = 3 independent experiments per drug concentration). Data analysis was carried out using GraphPad Prism version 9 (GraphPad Software, Inc.). Statistical analysis was performed using ordinary 2-way ANOVA. A p-value of less than 0.05 was considered statistically significant.

### Immunocytochemistry

H1 cells were seeded at a density of 1.2 × 10^4^ per well (1.2 × 10^5^ cells/mL, 100 µL per well) into 8-well µ-slides (Ibidi GmbH, Gräfelfing, Germany, cat. no. 240226) and allowed to adhere overnight. After 24 h, the wells received 100 µL of thioridazine in culture medium at 2 x experimental concentrations (15 µM), resulting in a final concentration of 7.5 µM. Control wells received 100 µL of fresh culture medium. The cells were then incubated for 72 h.

Following treatment, the culture medium was removed, and the cells were washed twice with cold PBS. Immediately after washing, the cells were fixed in 4% paraformaldehyde (PFA) in PBS for 10 min at RT and washed three times with PBS. To block non-specific antibody binding and permeabilize membranes, cells were incubated for 30 min at RT in blocking buffer consisting of 5% goat serum and 0.1% Triton X-100 in PBS, followed by three additional PBS washes.

Primary antibodies against three target proteins were diluted in blocking buffer according to the manufacturer´s recommendations or optimized conditions. The primary antibodies used were: rabbit-LC3A/B (1:100; Cell Signaling, cat. no. 12741 S), mouse-p62 (1:500; Abcam, cat. no. ab56416), and mouse-LAMP-2 (1:100; Abcam, cat. no. ab25631). Single-antibody controls were included for signal validation and used to justify the concentrations used in the primary antibody cocktail. Antibodies were diluted in blocking buffer at the specific ratios, and 100 µL of the antibody cocktail was added to each well. Slides were then incubated overnight at 4 °C.

The next day, the wells were washed three times with TBS-T, then incubated for 1 h at RT in the dark with 100 µL of a cocktail of fluorophore-conjugated secondary antibodies, each specific to its corresponding primary antibody: Alexa Fluor 647 goat anti-rabbit (Thermo Fisher Scientific, cat. no. A21244; working solution: 4 µg/mL from a 2 mg/mL stock), and Alexa Fluor 555 goat anti-mouse (Thermo Fisher Scientific, cat. no. A21422; working solution: 2 µg/mL from a 2 mg/mL stock).

After incubation, cells were washed three times with TBS-T. Nuclear staining was performed using a 300 nM DAPI solution, prepared by diluting a 14.3 mM stock 1:67 in PBS (yielding 300 µM) and then making a 1:1000 dilution to reach the final concentration. Cells were incubated with DAPI for 5 min in the dark, followed by three washes with PBS. Slides were cured overnight in the dark before imaging using a Leica Stellaris confocal microscope (Leica Microsystems GmbH). All imaging parameters were kept constant across samples for comparative analysis.

### Live cell fluorescence imaging

To obtain images of lysosomal compartments, cells were imaged without fixation, in contrast to the previous ICC. H1 cells were seeded at a density of 1.2 × 10^4^ per well (1.2 × 10^5^ cells/mL, 100 µL in each well) into 8-well µ-slides (Ibidi GmbH, Gräfelfing, Germany, cat. no. 240226) and allowed to adhere overnight. The following day, cells were either left as untreated controls or received thioridazine at time points of 4 h, 24 h, or 72 h prior to imaging. Treatment wells received 100 µL of thioridazine in culture medium at 2 x experimental concentrations (15 µM), resulting in a final concentration of 7.5 µM. Control wells received 100 µL (total volume 200 µL) of fresh culture medium. The cells were then incubated for 72 h.

Prior to imaging, LysoTracker Red DND-99 (Thermo Fisher Scientific) was diluted in culture medium to prepare a working solution as recommended by the manufacturer. Cells were incubated with the LysoTracker containing working solution for 1 h before imaging using a Leica Stellaris confocal microscope (Leica Microsystems GmbH). All imaging parameters were kept constant across samples for comparative analysis.

## Results

### MBM cell viability is decreased after thioridazine treatment

**Fig. 1:**
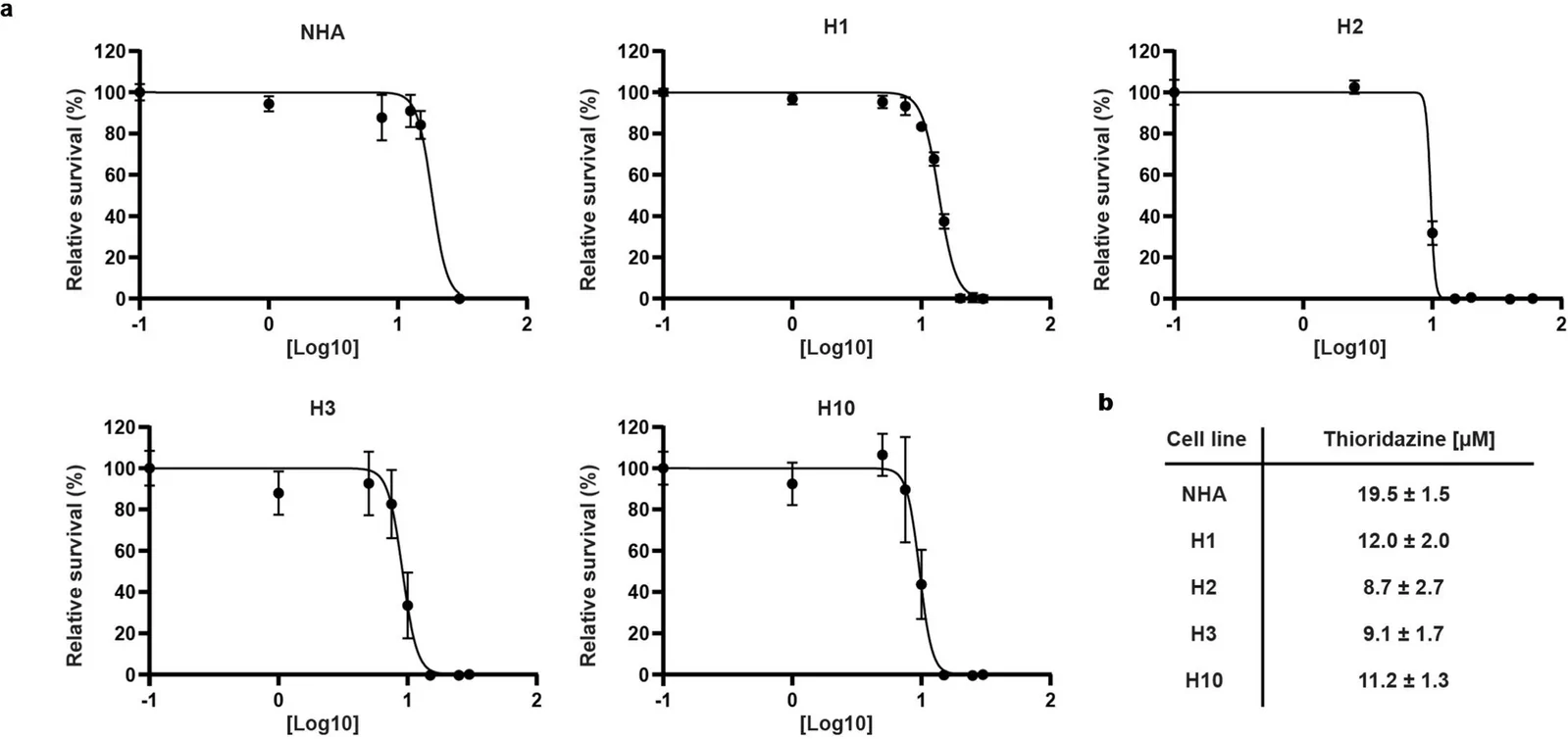
Thioridazine treatment reduces the viability of NHA and MBM cell lines in vitro in a dose-dependent manner. (**a**) Representative viability curves of cells grown in monolayers and exposed to increasing concentrations of thioridazine (mean ± SD). (**b**) Summary table showing the calculated mean IC_50_ doses with standard deviations for the control NHA cell line and the MBM cell lines (*N* = 3 independent experiments)

To establish whether thioridazine exerts cytotoxic effects on MBM cells, we conducted monolayer cell viability assays using patient-derived MBM cell lines H1, H2, H3, and H10. Normal human astrocytes (NHA) were included as controls to assess potential cytotoxic effects on normal brain cells. A dose-dependent decrease in the number of viable cells was observed following thioridazine treatment over 72 h. The half-maximal inhibitory concentration (IC_50_), defined as the dose at which 50% of the cells were no longer viable, was calculated for H1 (12.0 µM), H2 (8.7 µM), H3 (9.1 µM), and H10 (11.2 µM). In contrast, the IC_50_ for NHA cells was 19.5 µM, indicating a potential therapeutic window of 7.5–10.8 µM (Fig. 1, Supplementary Materials 1,2).

**Fig. 2:**
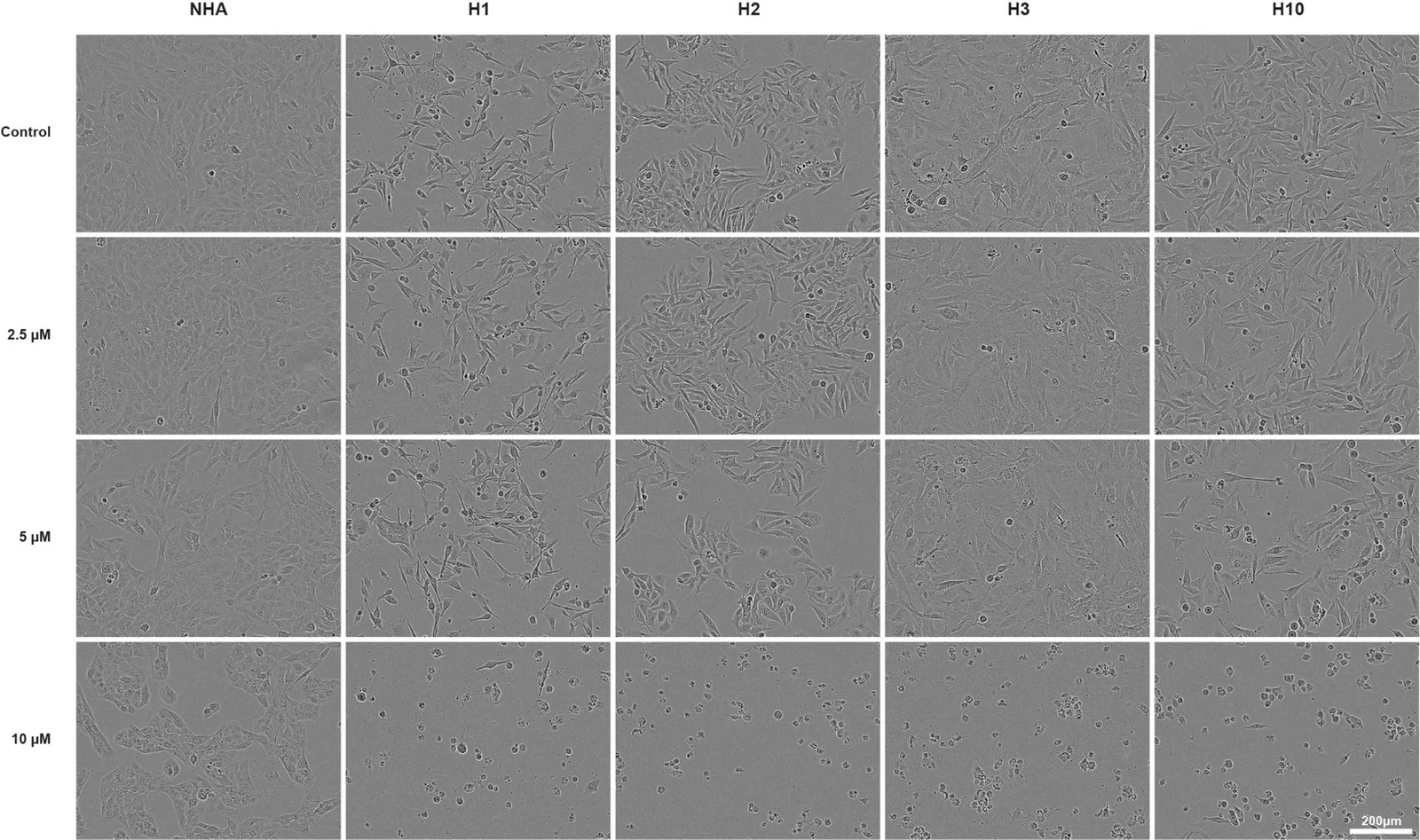
Thioridazine alters MBM cell morphology. Phase-contrast microscopic images of MBM cell lines (H1, H2, H3, and H10) and NHA in response to thioridazine exposure for 72 h at increasing doses (0 µM, 2.5 µM, 5 µM, and 10 µM). Scale bar = 200 μm

A light microscopy-based morphology study was performed to further support our findings. MBM cells (H1, H2, H3, and H10) together with NHA cells were treated with thioridazine at increasing doses (2.5, 5, and 10 µM). With increasing thioridazine concentrations, cells lost their spindle-like morphology and transformed into small spherical structures consistent with cell death (Fig. 2).

**Fig. 3:**
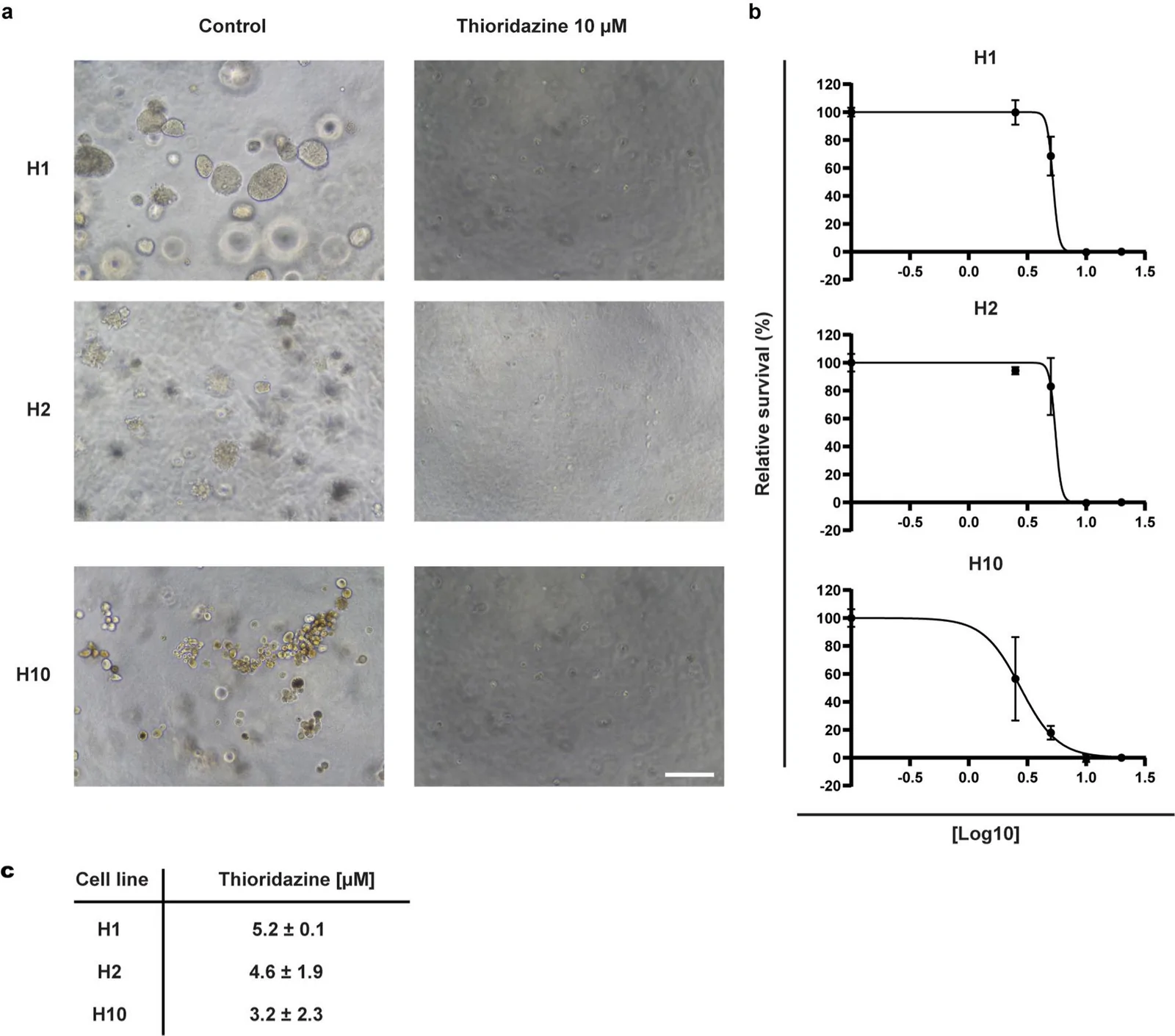
Thioridazine inhibits anchorage-independent cell viability in MBM cell lines. **(a**) Representative phase-contrast images of MBM cell lines (H1, H2, and H10) grown in soft agar, either untreated or treated with 10 µM thioridazine for 72 h, followed by incubation in drug-free medium for 21 days. Scale bar = 200 μm. (**b**) Representative viability curves following 72 h of thioridazine treatment at increasing doses (0 µM, 2.5 µM, 5 µM, 10 µM, 15 µM, 20 µM, 40 µM). Data are plotted as a percentage of the colorimetric density of the control well after 72 h of culture (relative survival, %). The IC_50_ is defined as the concentration corresponding to 50% relative survival. (**c**) Summary table showing the calculated IC_50_ doses with standard deviations for the MBM cell lines (H1, H2, and H10) (*N* = 3 independent experiments)

To assess the effects of thioridazine on colony formation and anchorage-independent cell growth, we performed a three-dimensional (3D) soft agar-based assay. MBM cells were cultured in a mixture of medium and agar, providing a scaffold supporting vertical and anchorage-independent growth. The IC_50_ values for the H1 (5.2 µM), H2 (4.6 µM), and H10 (3.2 µM) cell lines in the 3D assay (Fig. 3) were lower than those observed in the monolayer assays (Supplementary Material 3). However, this model did not support sufficient growth of the H3 and NHA cell lines.

**Fig. 4:**
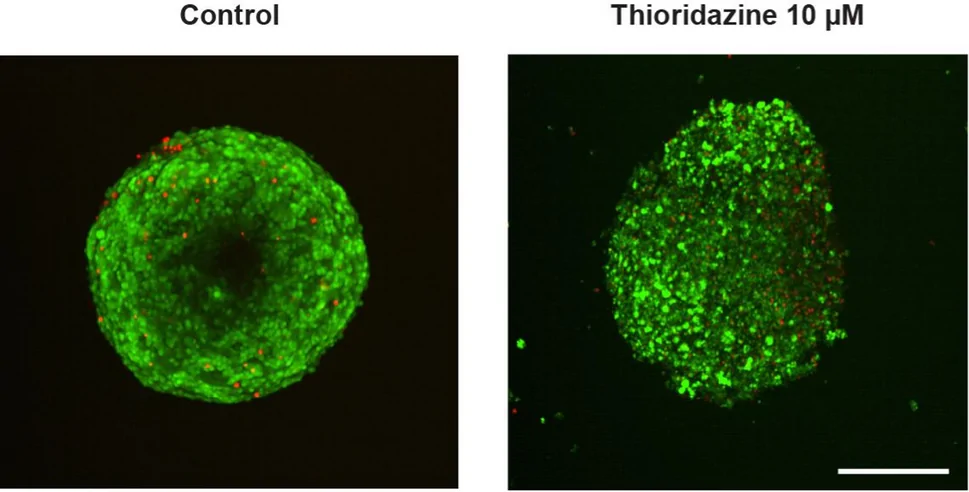
Thioridazine is not cytotoxic to fetal rat brain organoids (FRBOs) at doses corresponding to the MBM cell IC_50_ range. Representative confocal images showing live/dead staining of FRBOs after treatment with thioridazine for 72 h at a concentration of 10 µM, compared to an untreated control. Scale bar = 200 μm

After confirming the cytotoxic effects of thioridazine on MBM cells, we next investigated the potential impact of thioridazine on surrounding normal brain tissue. While NHA cells may serve as an initial screening tool for assessing drug effects, they do not fully mimic the complexity of the brain microenvironment. Thus, we employed fetal rat brain organoids (FRBOs), which contain all neuronal and glial cell populations within the brain [44]. After 72 h of treatment, FRBOs were stained using a live/dead viability kit and imaged by confocal microscopy. Untreated controls and 10 µM thioridazine-treated FRBOs were compared, with live cells appearing green and dead cells red. No increase in dead (red) cells was observed following thioridazine treatment (Fig. 4).

### Thioridazine inhibits MBM cell proliferation and migration in vitro

**Fig. 5:**
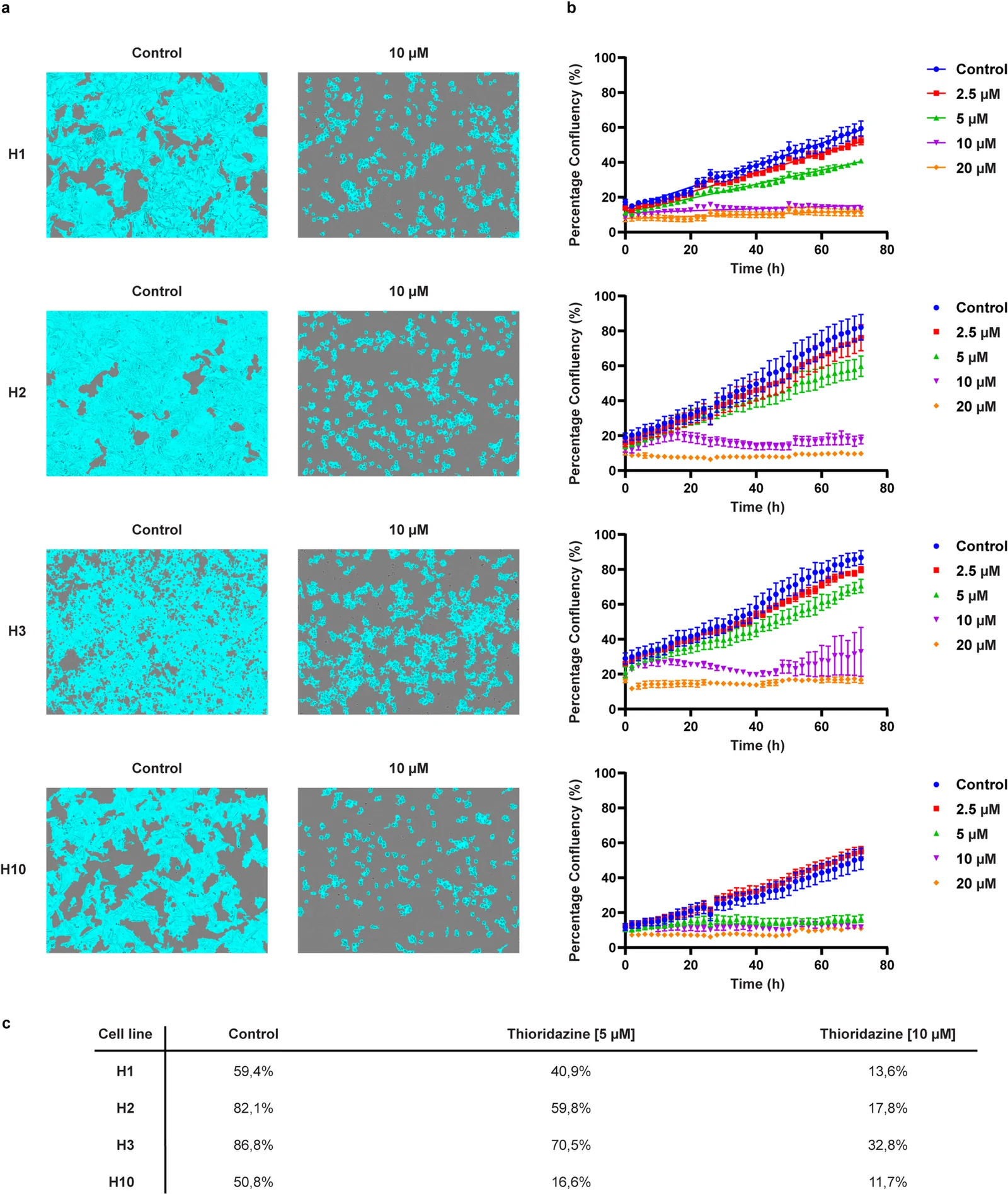
Thioridazine inhibits proliferation of MBM cell lines in vitro. (**a**) Representative light microscopy images of MBM cells cultured for 72 h in 96-well plates in either culture medium (control) or medium containing 10 µM thioridazine. Cell areas are visualized in light blue using an AI-assisted masking tool. (**b**) Representative graphs showing cell confluency at increasing thioridazine concentrations (0, 2.5, 5, 10, and 20 µM) during 72 h. Confluency was calculated as a percentage of the well area covered by cells. (c) Table showing the average confluency of MBM cell lines (H1, H2, H3, and H10) as a function of thioridazine concentration (0, 5, and 10 µM; *N* = 3 independent experiments). A full overview of the raw data used to produce these figures is available in the Supplementary Materials 4–15

To assess the real-time proliferative capacity of MBM cells after treatment with thioridazine, we used the IncuCyte live-cell imaging system. Cells were seeded at low density and treated for 72 h with increasing drug concentrations (2.5, 5, 10, or 20 µM) while being imaged every 2 h. Cell proliferation was quantified based on images of well confluency during this time period. For the H10 cell line, proliferation was completely inhibited at doses of 5 µM and above. For the remaining three cell lines, complete inhibition was observed at 10 and 20 µM (Fig. 5a, b). Thioridazine showed a dose- and time-dependent reduction in MBM cell proliferation, with marked differences between untreated controls and thioridazine-treated cells after 72 h (Fig. 5c).

Invasion and metastasis are well-established hallmarks of cancer, and cancer cell migration is an important process underlying these events [45]. Therefore, we performed a scratch wound assay to study the migratory capacity of MBM cells over 72 h using increasing concentrations of thioridazine (2.5, 5, 10, and 20 µM). A linear scratch was made in each semi-confluent MBM monolayer, generating a cell-free area into which the cells could migrate. Wound closure was used as a measure of migratory capacity.

**Fig. 6:**
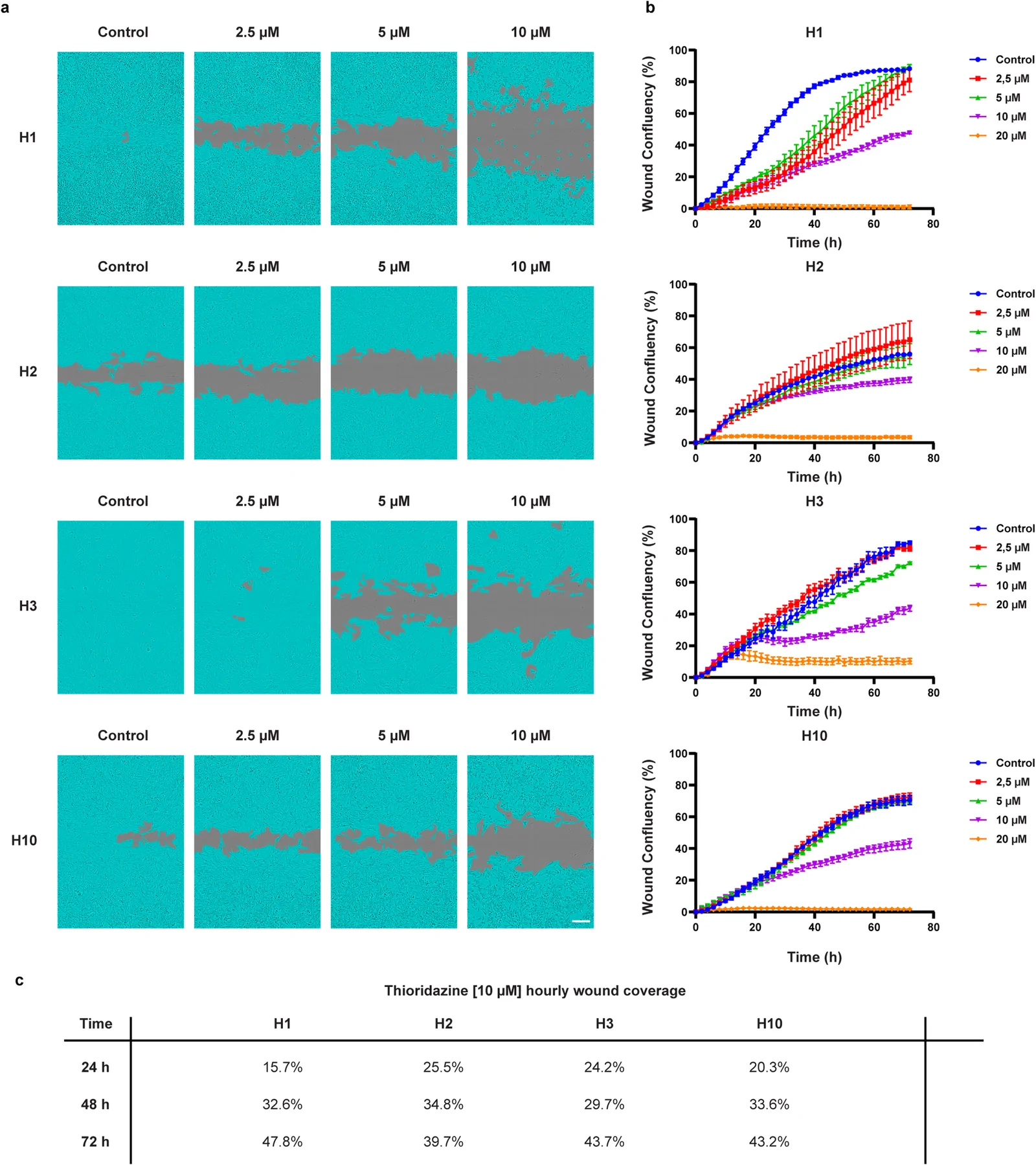
Thioridazine inhibits MBM cell migration in vitro. (**a**) Representative light microscopy images showing wound confluency after 72 h of treatment with increasing doses of thioridazine (0, 2.5, 5, and 10 µM). Cell areas are visualized in light blue using an AI-assisted masking tool. Scale bar = 200 μm. (**b**) Representative time-course graphs showing wound confluency over 72 h in response to thioridazine treatment. (**c**) Summary table showing the average confluency of MBM cell lines (H1, H2, H3, and H10) after 24, 48, and 72 h of treatment with 10 µM thioridazine (*N* = 3 independent experiments). A full overview of the raw data used to produce these figures is available in the Supplementary Materials 16–27

For all four cell lines, thioridazine inhibited wound closure in a dose-dependent manner (Fig. 6a, b). Quantification of wound confluency at 72 h showed that treatment with 10 µM thioridazine resulted in a wound confluency of around 48% for H1, 40% for H2, 44% for H3, and 43% for H10 cells (Fig. 6c).

### Anchorage-dependent colony formation is decreased after thioridazine treatment

**Fig. 7:**
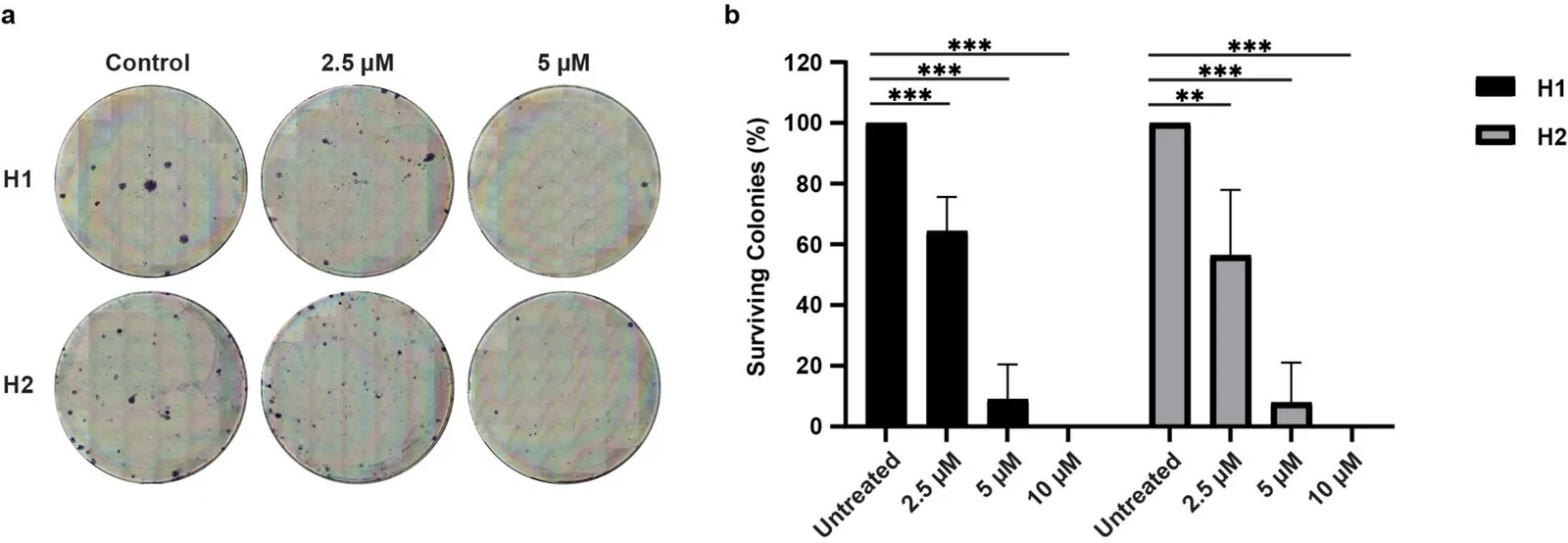
Thioridazine inhibits in vitro colony formation. (**a**) Representative light microscopy images of MBM cell colonies (H1 and H2) after 72 h of treatment with increasing doses of thioridazine (0, 2.5, and 5 µM), followed by growth in thioridazine-free medium for 14 days and subsequent staining with crystal violet. (**b**) Graphical representation of colony formation, showing the percentage of surviving MBM cell colonies (H1 and H2) following thioridazine treatment (2.5, 5, or 10 µM), relative to normalized untreated controls (100%) (Ordinary one-way ANOVA, ** = *P* < 0.01, *** = *P* < 0.001, *N* = 3 independent experiments). A full overview of the raw data used to produce the quantitative figures is available as Supplementary Material 28

To assess the effects of thioridazine on the clonogenic potential of H1 and H2 cells, anchorage-dependent colony formation assays were carried out. Cells were seeded at low density in 6-well culture plates, treated with thioridazine (2.5, 5, or 10 µM) or left as untreated controls receiving culture medium only for 72 h. After treatment, the plates were left to form colonies in drug-free culture medium for 14 days. The resulting colonies were fixed and stained with crystal violet (Fig. 7a). Thioridazine significantly inhibited colony formation of MBM cells in a dose-dependent manner, with clusters of more than 50 cells counted as colonies (Fig. 7a, b). Notably, at a concentration of 10 µM, no colonies were observed in either the H1 or H2 cell lines.

### Thioridazine does not consistently induce apoptosis in MBM cells

**Fig. 8:**
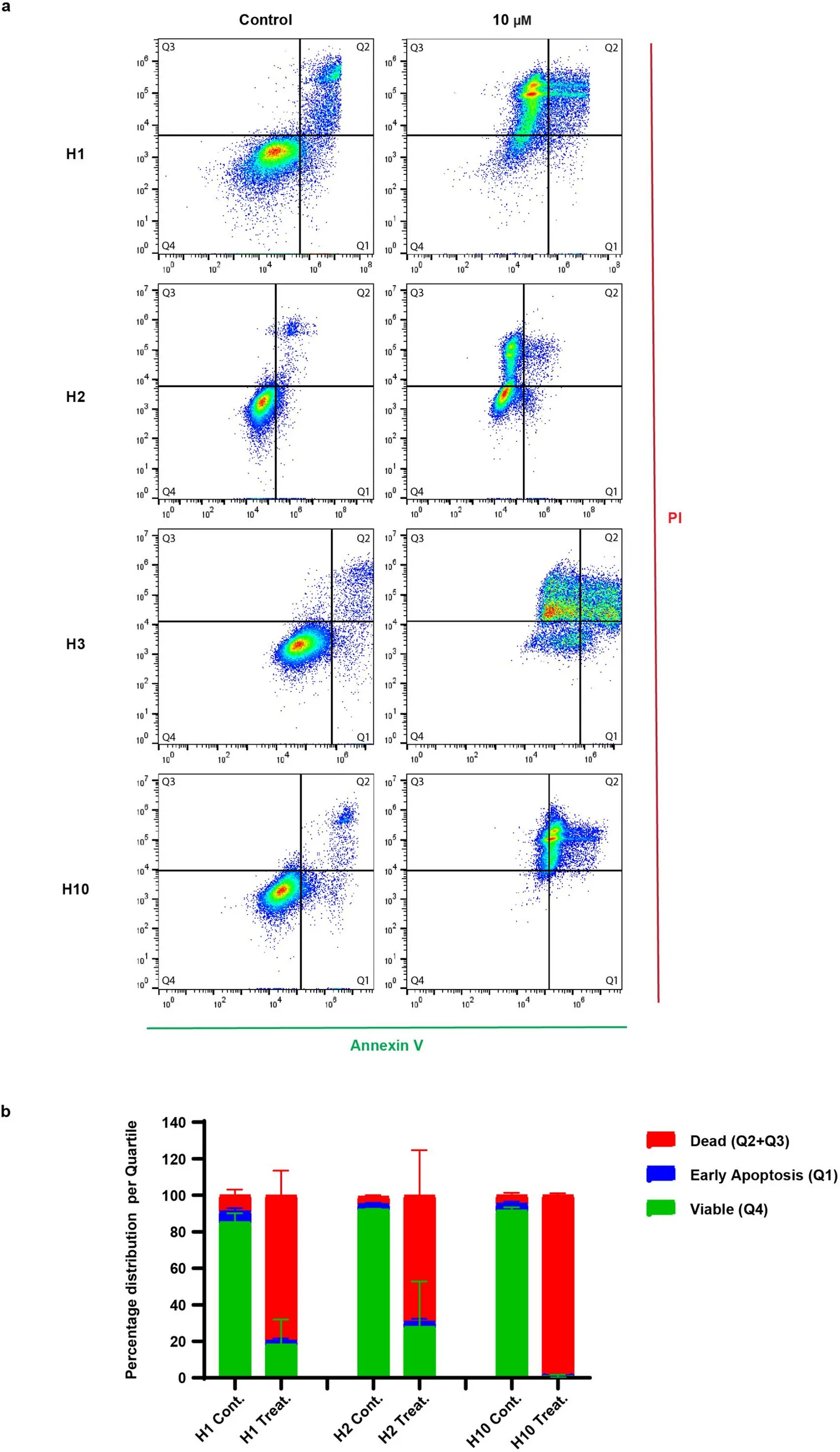
Flow cytometric analysis of apoptosis after treatment with thioridazine. (**a**) Representative dot plots showing the response of MBM cell lines (H1, H2, H3, and H10) treated with 10 µM thioridazine for 72 h compared to untreated cells. Annexin V labels apoptotic cells, while propidium iodide (PI) labels necrotic cells. Quadrant interpretation: Lower left (Q4) – viable cells, lower right (Q1) – early apoptotic cells, upper right (Q2) – late apoptotic cells, upper left (Q3) – necrotic or late apoptotic cells. The gating strategy is shown in Supplementary Material 30. (**b**) Bar graph showing the percentage distribution of cell populations in different quadrants representing different stages of apoptosis and necrosis. Upper quadrants (Q2 and Q3) were combined to represent late-stage cell death. The graphs compare MBM cell lines treated with thioridazine (10 µM, 72 h) with untreated controls and show the average distribution across experiments (*N* = 3 independent experiments). Extended flow cytometry graphs showing results for all cell lines (H1, H2, H3, and H10) with an extended range of thioridazine concentrations (0, 2.5, 5, 10 and 20 µM) can be viewed in Supplementary Material 31. A full overview of the exported raw data used to produce the quantitative figures is available as Supplementary Material 32

Having confirmed the inhibitory and cytotoxic effects of thioridazine and established effective working concentrations, we next sought to elucidate the underlying molecular mechanisms. Specifically, we studied whether thioridazine induced apoptosis using flow cytometric analysis. MBM cell lines (H1, H2, H3, and H10) were treated with thioridazine (2.5, 5, 10, and 20 µM) or left untreated for 72 h. Cells were then stained with Annexin V and propidium iodide (PI) and analyzed by flow cytometry (Fig. 8).

Thioridazine treatment induced late apoptosis/necrosis in all cell lines at 10 µM after 72 h, but did not have a clear effect at an earlier time point (24 h, Supplementary Material 29). Notably, a large proportion of cells showed staining with both dyes, suggesting a progression towards both late apoptosis (Fig. 8, Q2) and necrosis (Fig. 8, Q3). These findings suggest that MBM cells treated with thioridazine progress into late-stage cell death, with limited accumulation in apoptotic stages.

### Exploring the subcellular effects of thioridazine

To further investigate the molecular effects underlying thioridazine-induced cell death, we performed Western blot analyses of key apoptosis-related proteins. The pro-apoptotic markers cleaved PARP1 and cleaved caspase-3, as well as the anti-apoptotic protein Bcl-2, showed variable expression patterns across the MBM cell lines.

**Fig. 9:**
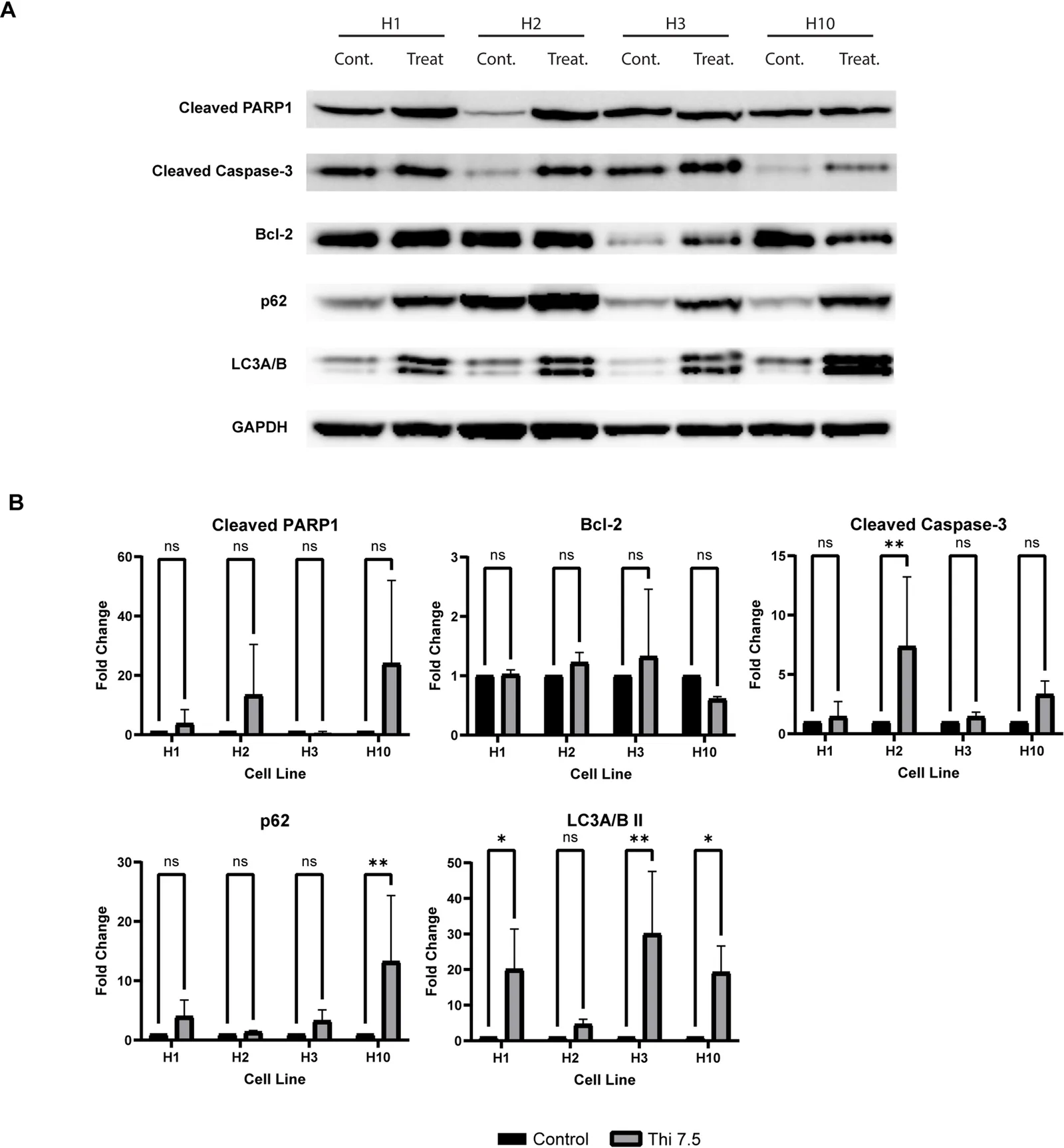
Thioridazine does not consistently upregulate apoptosis-related proteins in MBM cells. (**a**) Western blots of the MBM cell lines (H1, H2, H3, and H10) treated with thioridazine (7.5 µM for 72 h), showing the expression levels of the pro-apoptotic proteins cleaved PARP1 and cleaved caspase-3, the anti-apoptotic protein Bcl-2, and the autophagy-associated markers p62 and LC3A/B. (**b**) Graphical representation of the densitometric quantifications of the western blots showing the fold change in protein expression in treated samples relative to untreated controls. GAPDH-normalized band intensities were quantified using ImageJ/Fiji. (Ordinary two-way ANOVA, ns = *P* > 0.05, * = *P* < 0.05, ** = *P* < 0.01, *N* = 3 independent experiments). Complete blots and their corresponding loading controls are shown in Supplementary Materials 33–39. Baseline caspase-3 expression in the respective cell lines (H1, H2, H3, and H10) following 72 h incubation with or without 7.5 µM thioridazine are shown in Supplementary Materials 40 and 41

Cleaved PARP1 levels increased in H1 and H2 cells following treatment but remained unchanged in H3 and H10 cells. Further, cleaved caspase-3 levels increased in H2 and H10 cell lines after exposure to 7.5 µM thioridazine but remained unchanged in H1 and H3 cell lines. Bcl-2 expression levels increased in H3, decreased in H10, and showed no changes in H1 and H2 cell lines (Fig. 9).

Inhibition of autophagic flux could contribute to the observed inhibitory and cytotoxic effects of thioridazine on MBM cells in vitro. To study this, we performed Western blot analyses of two key autophagy-associated markers: p62 and microtubule-associated protein 1 light chain 3 alpha and beta (LC3A/B). Both proteins showed increased expression in all four MBM cell lines (H1, H2, H3, and H10) following exposure to 7.5 µM thioridazine for 72 h (Fig. 9).

Taken together, our findings demonstrate heterogeneous responses across the four MBM cell lines, suggesting that thioridazine does not consistently activate canonical apoptotic signalling pathways. Simultaneously, the consistent accumulation of p62 and LC3A/B indicates impaired autophagic degradation and inhibited autophagic flux. This may, at least in part, explain the observed inconsistencies in apoptosis, as disrupted autophagic flux may interfere with canonical apoptotic pathways.

The effects of thioridazine on lysosomes, a key component of autophagic flux, warrant further investigation given the apparent interaction between thioridazine and autophagy. To further assess whether thioridazine halts autophagic flux or directly stimulates autophagosome production, we performed autophagic flux experiments utilizing Bafilomycin A1 (BAFA1) in combination with thioridazine. Our results show that BAFA1 does not increase LC3 or p62 expression in combination with thioridazine (Supplementary Materials 42 and 43).

**Fig. 10:**
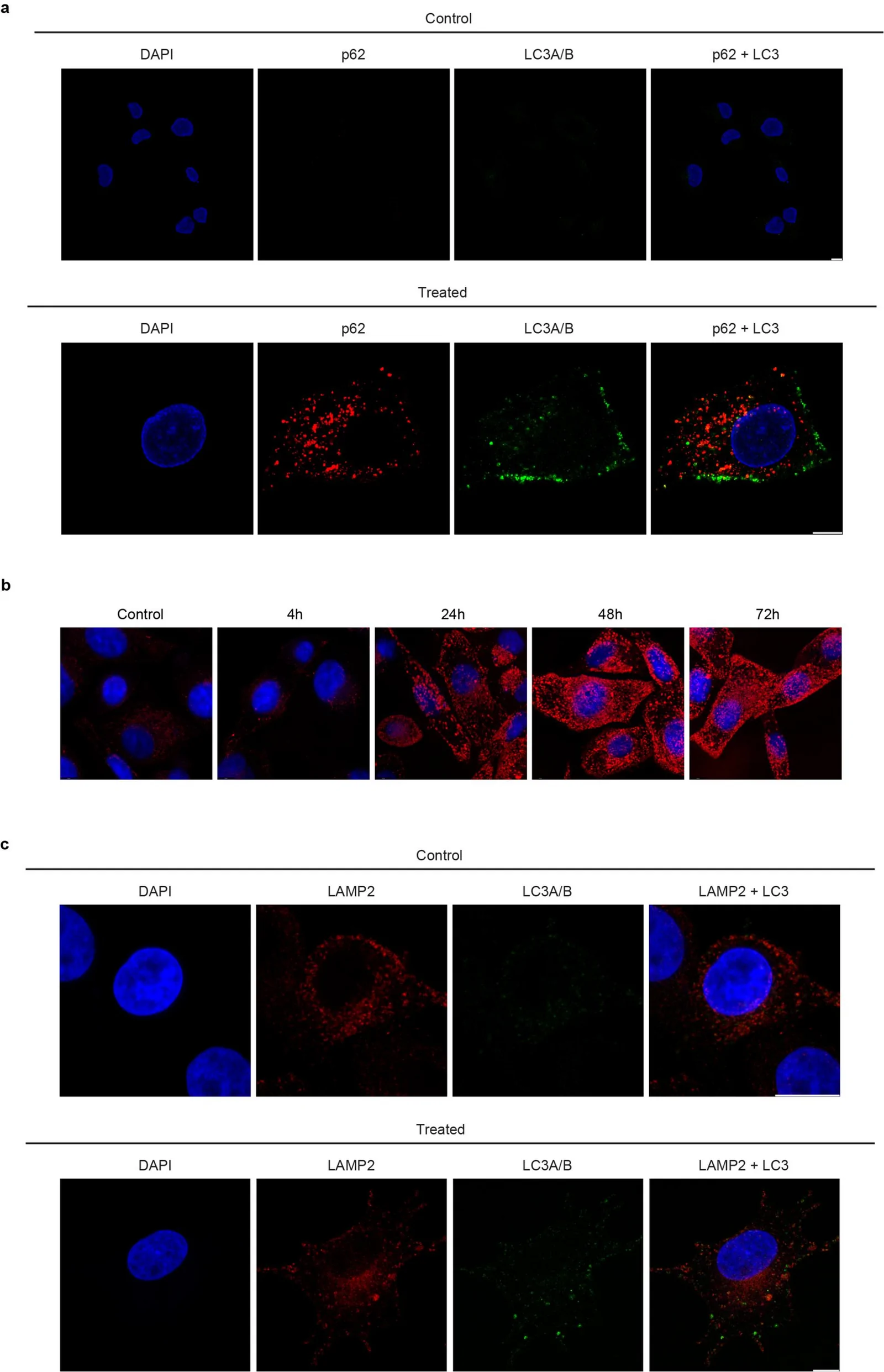
Autophagy markers and lysosomes accumulate intracellularly in H1 cells following thioridazine treatment. (**a**) Confocal images stained for DAPI (blue), p62 (red), and LC3A/B (green), followed by a composite image of all three channels. Results are shown for untreated control cells and cells treated with 7.5 µM thioridazine for 72 h. Images are maximum-intensity projections. (**b**) Confocal images of H1 cells treated with thioridazine for up to 72 h (4, 24, 48, and 72 h), showing nuclei stained with DAPI (blue) and lysosomes stained with LysoTracker (red). (**c**) Confocal images of H1 cells stained for DAPI (blue), LAMP-2 (red), and LC3A/B (green), followed by a composite image of all three channels. Results are shown for untreated control cells and cells treated with 7.5 µM thioridazine for 72 h. Images shown are representative and intended to qualitatively illustrate the intracellular localization of autophagy- and lysosome-associated markers in support of the Western blot analyses (*N* = 1)

Previous research on primary brain cancer cell lines has reported divergent effects on lysosomes. Johannesen et al. demonstrated that thioridazine effectively reduces the presence of lysosomes in treated cells, whereas Zhou et al. reported an increase in lysosomal abundance following thioridazine treatment [14, 21]. Therefore, to investigate the spatial distribution of the autophagy markers p62 and LC3A/B and to assess the effects of thioridazine on lysosomal abundance in MBM cells, we performed immunocytochemistry (ICC) (Fig. 10). Consistent with the increased levels of p62 and LC3A/B observed by Western blotting (Fig. 9), thioridazine treatment resulted in marked intracellular accumulation of both p62 and LC3A/B, with a predominantly punctate staining pattern compared with untreated control cells (Fig. 10a).

Time-course staining using LysoTracker revealed apparent morphological alterations and an increase in lysosomal signal intensity over the course of thioridazine treatment, becoming more pronounced at later time points (24–72 h) (Fig. 10b). In line with this observation, co-staining for the lysosomal membrane protein LAMP-2 and LC3A/B demonstrated increased intracellular lysosomal structures in thioridazine-treated cells, with partial spatial overlap between LC3A/B-positive structures and LAMP-2-positive compartments (Fig. 10c).

Together, these qualitative imaging data support the biochemical findings and suggest that thioridazine treatment is associated with intracellular accumulation of autophagy- and lysosome-associated markers, consistent with impaired autophagic flux in MBM cells.

## Discussion

Recent years have seen considerable advances in the treatment of melanoma. Immunotherapies such as inhibitors of programmed cell death protein 1 (PD-1) and cytotoxic T-lymphocyte-associated antigen 4 (CTLA-4) are now cornerstones in clinical practice and constitute first-line treatment for many patients with metastatic melanoma. Patients with non-brain metastatic melanoma generally respond well to combination immunotherapy, with studies showing that median overall survival (OS) is not reached even after 60 months of follow-up [46].

The clinical reality is different for patients with MBM, where responses to immunotherapy and OS largely depend on factors such as symptom burden at diagnosis, the need for corticosteroid treatment, and the presence of leptomeningeal disease. Several large-scale, long-term follow-up studies have demonstrated that patients exhibiting any of the aforementioned factors experience significantly lower response rates and overall survival [3–5]. Consequently, current European clinical guidelines are cautious about initiating first-line immunotherapy in patients with symptomatic disease [47].

Targeted inhibitors represent the next available systemic treatment options for these patients. BRAF mutations are the most common genetic alterations found in MBM, occurring in approximately half of melanoma patients [7]. Consequently, small-molecule inhibitors targeting BRAF and its downstream effector MEK have been extensively evaluated in the clinic and have shown intracranial response rates of approximately 44–59%, depending on BRAF mutation type (V600 E/K/R), Eastern Cooperative Oncology Group (ECOG) performance status, symptom burden, and previous lines of treatment. However, the median duration of the intracranial response rate is low, ranging from only 4.5 to 8.3 months [6]. Thus, while BRAF/MEK inhibitors can provide transient disease control, patients who relapse following targeted treatments, particularly those who have already progressed on or are ineligible for immunotherapy, have few remaining systemic treatment alternatives outside of clinical trials.

In the current work, we investigated the potential of repurposing thioridazine, a clinically established antipsychotic, as an adjuvant treatment option for MBM patients. This strategy was motivated by previous reports demonstrating inhibitory effects of thioridazine in cell cultures of primary brain tumors [14, 21, 26] as well as non-brain metastatic melanoma cell lines [19, 28].

Our viability experiments revealed a small yet exploitable therapeutic window, with the IC_50_ dose in NHA cells being approximately two times higher than that observed in our MBM cell lines (Fig. 1). The IC_50_ values found in our study are comparable to previous reports in glioblastoma [14, 21] and melanoma cell lines [28]. Furthermore, increasing concentrations of thioridazine induced pronounced morphological changes in the MBM cell lines, observed as loss of spindle-like features and progression toward spherical structures with reduced cell volume. These changes occurred in line with observed reductions in cell viability (Fig. 2).

Anchorage-independent (3D) viability experiments showed comparable doses to those found in monolayer cultures, although generally at somewhat lower concentrations (Fig. 3). Discrepancies between monolayer (2D) and three-dimensional spheroid-based assays have been reviewed previously and are likely due to differences in drug diffusivity, mechanisms of drug action, and cell proliferation capabilities [48]. In our laboratory, however, we have previously reported similar results, showing that the IC_50_ doses decline when using a three-dimensional soft agar model, potentially reflecting reduced cell-cell interactions and the growth of isolated colonies [13].

To further investigate the effects of thioridazine on healthy brain tissue, we utilized the FRBO model system, which mimics the cellular complexity of the healthy brain better than a 2D system. At concentrations corresponding to the IC_50_ values observed in MBM cells (10 µM), no cell death was detected in the FRBOs as assessed by Live/Dead staining (Fig. 4) [49]. These findings support the presence of a therapeutic window in the context of brain tissue exposure. However, it is possible that the structural changes observed in our FRBOs following thioridazine treatment may reflect underlying cellular, biological, or biochemical alterations that are not captured by the Live/Dead staining assay.

Sustaining proliferative signaling is a key hallmark of tumor biology, and current literature indicates that proliferation is increased in many MBM cell lines compared with healthy tissues [45, 50, 51]. We show that thioridazine, at concentrations of 5 µM and above, clearly inhibits MBM cell proliferation in a dose-dependent manner (Fig. 5). Similarly, cell migration was inhibited at comparable doses (Fig. 6). Together, these findings suggest that thioridazine exerts anti-tumoral effects in MBM cells even at concentrations below the estimated IC_50_ values.

Tumor cell migration is of particular clinical relevance, as local invasion is a critical factor in melanoma staging and in the development of metastases [52]. Therefore, inhibition of tumor cell migration is an important characteristic of potential therapeutic agents for melanoma treatment. Furthermore, the ability of single melanoma cells to form colonies and develop into clinically relevant tumors presents a significant challenge in the context of micrometastases [53]. We show that thioridazine inhibits this capability in MBM cell lines in a dose-dependent manner (Fig. 7). Our findings are supported by previous reports demonstrating similar dose-dependent inhibition of colony formation following thioridazine treatment in other brain cancers [21].

Although we have mapped thioridazine’s overarching anti-tumoral effects in MBM cells, its mechanism of action in this context remains unresolved. Previous studies describe a wide range of proposed mechanisms underlying the anti-tumoral effects of thioridazine in other cancer types, one of which involves disruption of DNA damage response pathways, specifically through inhibition of ataxia telangiectasia mutated (ATM) and Rad3-related protein (ATR), and activation of checkpoint kinase 1 (Chk1) [24, 25]. In addition, thioridazine has been widely studied in combination with other chemotherapeutic agents, and its potential to inhibit the efflux pump P-glycoprotein has been hypothesized to enhance intracellular drug accumulation [41]. However, other mechanisms are more extensively documented.

Apoptosis, or programmed cell death, remains the best-characterized and clinically targeted form of tumor cytotoxicity in oncology [54] and has been the focus of previous studies on phenothiazines in cancer, including melanoma [19, 27]. Thus, we examined apoptotic responses following thioridazine treatment in MBM cells. Our flow cytometry analyses, however, suggest that thioridazine treatment leads to a shift from viable cells directly into loss of membrane integrity with strong PI staining (Fig. 8). Annexin V positivity only emerges alongside increased PI signal and likely reflects late-stage necrosis, exposing intracellular phosphatidylserine (PS) for annexin V binding rather than canonical apoptosis [55].

This interpretation is supported by our Western blot analysis of established apoptotic markers in MBM cells following thioridazine treatment (Fig. 9). At a concentration of 7.5 µM, thioridazine elicited heterogeneous responses in apoptosis markers across the MBM cell lines. For example, the H1 and H2 cell lines showed increased levels of pro-apoptotic markers (cleaved PARP1 and cleaved caspase-3) with stable anti-apoptotic marker expression (Bcl-2), while the H3 cell line showed the opposite effect. Interestingly, the H3 cell line contains a BRAF mutation (BRAF L577F) that has, to our knowledge, not previously been reported in the literature. The unique mutational status of this cell line could perhaps explain some of the aberrant responses we see in comparison with our other, better documented, cell lines. As a result, even if apoptosis is involved, other mechanisms are likely also contributing to the anti-tumoral effects of thioridazine. Our observations are supported by other studies reporting no increase in cleaved caspase-3 or cleaved PARP1 in cells treated with thioridazine [14]. Notably, others have shown that thioridazine only induces apoptosis at concentrations well above the IC_50,_ with some suggesting that this apoptotic response could be p62-mediated and associated with impaired autophagic flux [19, 21, 26].

With the observed heterogeneity in apoptotic responses and the potential involvement of p62, our attention turned to autophagy as a putative cellular response to thioridazine treatment. Western blot analysis demonstrated a clear and reproducible increase in both p62 and LC3A/B after drug exposure (Fig. 9). This is consistent with other reports showing upregulation of LC3A/B and p62 in cells treated with comparable doses (7.5-8 µM) [14, 21].

When autophagic flux is intact, p62 binds ubiquitinated proteins and transports them to the forming autophagosome through interaction with LC3 at the autophagosomal membrane. p62 is subsequently degraded together with its bound cargo following autophagosome-lysosome fusion. Consequently, increased autophagic signaling is typically associated with increased LC3A/B levels combined with stable or reduced p62 expression. Interestingly, the simultaneous increase in both p62 and LC3A/B seen in our experiments indicates impaired autophagic flux due to accumulation of p62, consistent with previous findings in glioblastoma models treated with thioridazine [14]. However, alternative mechanisms have been reported, as Chu et al. found that the thioridazine-induced increase in p62 expression was linked to β-catenin degradation and not the result of impaired autophagic flux [26]. Our experiments show that co-treatment with thioridazine and bafilomycin A1 resulted in minimal additional accumulation of LC3 and p62 compared to thioridazine treatment alone (Supplementary Material 42). Since bafilomycin A1 is a known blocker of lysosomal degradation of autophagosomes, the absence of an additive effect in LC3 accumulation suggests that thioridazine may already impair autophagic flux at the level of autophagosome degradation [56].

Direct visualization of autophagy-related components represented a logical next step. Using confocal microscopy, we confirmed that thioridazine treatment increased p62 and LC3A/B levels in MBM cell lines (Fig. 10a). Limited co-localization between p62 and LC3 was seen, suggesting impaired macroautophagy. Furthermore, LC3A/B accumulated along the cell periphery, a pattern that, to our knowledge, has not been reported previously in the context of thioridazine treatment.

The peripheral accumulation of autophagosomes may reflect thioridazine’s described ability to accumulate in the cell membrane and compromise plasma membrane integrity and repair processes [39]. However, the observed subcellular distribution also fits well with a recently described non-lytic autophagic process called secretory autophagy [57]. In this process, loss of lysosomal integrity necessitates a cellular adaptation to maintain homeostasis. As a result, autophagosomes are directed toward the cell membrane to release their contents extracellularly [58].

The lysosomotropic properties of thioridazine have been described previously and provide further support for the notion that thioridazine may alter lysosomal function [59, 60]. In line with this, our results demonstrate that lysosomes in MBM cells increase in number and undergo morphological alterations following thioridazine exposure in a time-dependent manner (Fig. 10b). Moreover, we observed limited co-localization between lysosomes (LAMP-2) and autophagosomes (LC3A/B) in thioridazine-treated MBM cells (Fig. 10c), suggesting a relative lack of autophagosome–lysosome fusion. Together, these findings are consistent with the activation of an alternative autophagic pathway, such as secretory autophagy.

A limitation of the present study is that the findings are based on in vitro experiments. Thus, further studies in animal models will be required to confirm the therapeutic potential of thioridazine in MBM.

A clinical limitation, however, is that the use of thioridazine may be associated with dose-limiting side-effects such as slowed cognition, headache, retinotoxicity, and in rare cases cardiac arrhythmias [61–63]. Nevertheless, toxicity is described as rare at oral doses below 800 mg daily, establishing this as the recommended upper limit of daily intake [64]. Studies of steady-state thioridazine concentrations show that each milligram of oral thioridazine increases serum concentration by approximately 5 nM [65]. Further, studies show that thioridazine reaches brain concentrations at least five times higher than those found in serum [66]. With this in mind, 400 mg of oral thioridazine would give a theoretical serum concentration of 2 µM, which in turn corresponds to a 10 µM concentration in the brain parenchyma. Therefore, achieving brain concentrations of 10 µM thioridazine would theoretically be feasible even at the lower end of currently used clinical doses. Translation of thioridazine into clinical cancer treatment would likely occur in combination with other modalities. However, this lies beyond the scope of the present study.

Our study represents the first experimental exploration of thioridazine’s effects on MBM cell lines. Although our findings show broad aspects of apoptosis and autophagy induction, it is clear that further studies into non-apoptotic cell death, cell cycle regulation and mechanistic autophagy studies would be of great interest for further research.

## Conclusions

We demonstrate for the first time that thioridazine exerts anti-tumoral effects in MBM cell lines. Thioridazine reduced MBM cell viability and inhibited proliferation, migration, and colony formation in a dose-dependent manner in vitro, while showing minimal toxicity in FRBOs. Our data indicate that although a potential contributor, apoptosis does not play a major role in thioridazine-induced cytotoxicity. Instead, dysregulation of autophagy appears to be a significant component of thioridazine’s mechanism of action in MBM cells.

We propose that thioridazine induces increased autophagosome formation with p62 accumulation and altered lysosomal dynamics, resulting in enhanced autophagic signaling but impaired autophagic flux, potentially shifting the cells towards a form of secretory autophagy. These findings highlight the potential of thioridazine for therapeutic repurposing and warrant further investigation into the molecular mechanisms underlying the disrupted autophagic flux observed in MBM cell lines.

## Supplementary Information

Supplementary Material 1

## Data Availability

All data generated or analysed during this study are included in this published article and its supplementary information files.

## Abbreviations

AMPK − 5' AMP: activated protein kinase
ATR: Ataxia telangiectasia and Rad3-related
BBB: Blood-brain barrier
Bcl-2: B-cell lymphoma 2
BM: Brain metastases
BRAF: v-Raf murine sarcoma viral oncogene homolog B1
c-Caspase 3: Cleaved caspase 3
c-PARP1: Cleaved poly(ADP-ribose) polymarese 1
Chk1: Checkpoint kinase 1
CNS: Central nervous system
CSF: Cerebrospinal fluid
CTLA4: Cytotoxic T-lymphocyte-associated antigen 4
DRD2: Dopamine receptor D2
ECOG: Eastern Cooperative Oncology Group (Performance Status)
EDTA: Ethylenediamine tetraacetic acid
FRBOs: Fetal rat brain organoids
FZD: Frizzled receptors
GAPDH: Glyceraldehyde 3-phosphate dehydrogenase
GSK3β: Glycogen synthase kinase-3 beta
H1, H2, H3, H10: Patient derived melanoma brain metastases cell lines
ICC: Immunocytochemistry
IC50: Half maximal inhibitory concentration
LAG3: Lymphocyte-activation gene 3
LAMP-2: Lysosome-associated membrane protein 2
LC3A/B: Microtubule-associated protein 1 light chain 3 alpha and beta
MBM: Melanoma brain metastases
NHA: Normal human astrocytes
OS: Overall survival
PD1: Programmed cell death protein 1
PFA: Paraformaldehyde
PI: Propidium iodide
REC: The Regional Ethical Committee
RIPA: Radioimmunoprecipitation assay
RT: Room temperature
SQSTM1/p62: Sequestome 1
TBS: Tris-buffered saline
TBS: T-Tris buffered saline containing 0.1% Tween-20
TCTP: Translationally controlled tumor protein
ULK1: Unc-51-like autophagy activating kinase 1
WST: 1-Water soluble tetrazolium (salt) − 1
3D: Three dimensional

## References

[1] Folkehelseinstituttet [Internet]. 2025 [cited 2025 Jul 28]. Melanom (føflekkreft). Available from: https://www.fhi.no/kreft/nokkeltall/kreftformer/melanom/

[2] Zheng S, Lin Z, Zhang R, Cheng Z, Li K, Gu C, et al. Progress in immunotherapy for brain metastatic melanoma. Front Oncol. 2025;14. 10.3389/fonc.2024.1485532. PMC11810730 39935851

[3] Long GV, Atkinson V, Lo SN, Guminski AD, Sandhu SK, Brown MP, et al. Ipilimumab plus nivolumab versus nivolumab alone in patients with melanoma brain metastases (ABC): 7-year follow-up of a multicentre, open-label, randomised, phase 2 study. Lancet Oncol. 2025;26(3):320–30. 2045(24)00735-6 PubMed PMID: 39978375. 10.1016/S1470-2045(24)00735-6 39978375

[4] Tawbi HA, Forsyth PA, Hodi FS, Lao CD, Moschos SJ, Hamid O, et al. Safety and efficacy of the combination of nivolumab plus ipilimumab in patients with melanoma and asymptomatic or symptomatic brain metastases (CheckMate 204). Neuro-Oncol. 2021;23(11):1961–73. 10.1093/neuonc/noab094 . PubMed PMID: 33880555; PubMed Central PMCID: PMC8563325. PMC8563325 33880555

[5] Tawbi HA, Forsyth PA, Hodi FS, Algazi AP, Hamid O, Lao CD, et al. Long-term outcomes of patients with active melanoma brain metastases treated with combination nivolumab plus ipilimumab (CheckMate 204): final results of an open-label, multicentre, phase 2 study. Lancet Oncol. 2021;22(12):1692–704. 10.1016/S1470-2045(21)00545-3. PMC9328029 34774225

[6] Davies MA, Saiag P, Robert C, Grob JJ, Flaherty KT, Arance A, et al. Dabrafenib plus trametinib in patients with BRAF V600–mutant melanoma brain metastases (COMBI-MB): a multi-cohort, open-label, phase 2 trial. Lancet Oncol. 2017;18(7):863–73. 17)30429-1 PubMed PMID: 28592387; PubMed Central PMCID: PMC5991615. 10.1016/S1470-2045(17)30429-1 PMC5991615 28592387

[7] Cheng L, Lopez-Beltran A, Massari F, MacLennan GT, Montironi R. Molecular testing for BRAF mutations to inform melanoma treatment decisions: a move toward precision medicine. Mod Pathol. 2018;31(1):24–38. 10.1038/modpathol. 2017.104 PubMed Central PMCID: PMC5758899. PMC5758899 29148538

[8] Stanford University A, Multicenter. Phase II Trial of Relatlimab, Nivolumab, and Ipilimumab in Patients With Asymptomatic and Symptomatic Melanoma Brain Metastases [Clinical trial registration] [Internet]. clinicaltrials.gov; 2025 Apr [cited 2025 Jul 28]. Clinical trial registration no.: NCT06712927. Available from: https://clinicaltrials.gov/study/NCT06712927

[9] Atkins MB, Lee SJ, Chmielowski B, Tarhini AA, Cohen GI, Truong TG, et al. Combination Dabrafenib and Trametinib Versus Combination Nivolumab and Ipilimumab for Patients With Advanced BRAF-Mutant Melanoma: The DREAMseq Trial-ECOG-ACRIN EA6134. J Clin Oncol Off J Am Soc Clin Oncol. 2023;41(2):186–97. 10.1200/JCO.22.01763 . PubMed PMID: 36166727; PubMed Central PMCID: PMC9839305. PMC9839305 36166727

[10] Liu B, Zhou H, Tan L, Siu KTH, Guan XY. Exploring treatment options in cancer: tumor treatment strategies. Signal Transduct Target Ther. 2024;9(1):175. 10.1038/s41392-024-01856-7. PMC11252281 39013849

[11] Wieder R, Adam N. Drug repositioning for cancer in the era of AI, big omics, and real-world data. Crit Rev Oncol Hematol. 2022;175:103730. 10.1016/j.critrevonc.2022.103730. 35654244

[12] Wu D, Chen Q, Chen X, Han F, Chen Z, Wang Y. The blood–brain barrier: Structure, regulation and drug delivery. Signal Transduct Target Ther. 2023;8(1):217. 10.1038/s41392-023-01481-w. PMC10212980 37231000

[13] Wikerholmen T, Taule EM, Rigg E, Berle BF, Sættem M, Sarnow K, et al. Repurposing neuroleptics: clozapine as a novel, adjuvant therapy for melanoma brain metastases. Clin Exp Metastasis. 2025;42(2):12. 10.1007/s10585-025-10328-3 . PubMed PMID: 39856383; PubMed Central PMCID: PMC11761981. PMC11761981 39856383

[14] Johannessen TC, Hasan-Olive MM, Zhu H, Denisova O, Grudic A, Latif MA, et al. Thioridazine inhibits autophagy and sensitizes glioblastoma cells to temozolomide. Int J Cancer. 2019;144(7):1735–45. 10.1002/ijc.31912 . PubMed PMID: 30289977. 30289977

[15] Fenton M, Rathbone J, Reilly J, Sultana A. Thioridazine for schizophrenia. Cochrane Database Syst Rev. 2007;2007(3):Cd001944. 10.1002/14651858.CD001944.pub2 . PubMed Central PMCID: PMC6718212. PMC6718212 17636691

[16] PubChem. Thioridazine [Internet]. [cited 2025 Jul 29]. Available from: https://pubchem.ncbi.nlm.nih.gov/compound/5452

[17] Song Y, Li L, Chen J, Chen H, Cui B, Feng Y, et al. Thioridazine hydrochloride: an antipsychotic agent that inhibits tumor growth and lung metastasis in triple-negative breast cancer via inducing G0/G1 arrest and apoptosis. Cell Cycle. 2020;19(24):3521–33. 2020.1850969 PubMed Central PMCID: PMC7781661. 10.1080/15384101.2020.1850969 PMC7781661 33315498

[18] Tegowski M, Fan C, Baldwin AS. Thioridazine inhibits self-renewal in breast cancer cells via DRD2-dependent STAT3 inhibition, but induces a G(1) arrest independent of DRD2. J Biol Chem. 2018;293(41):15977–90. 10.1074/jbc.RA. 118.003719 PubMed Central PMCID: PMC6187640. PMC6187640 30131338

[19] Gil-Ad I, Shtaif B, Levkovitz Y, Nordenberg J, Taler M, Korov I, et al. Phenothiazines induce apoptosis in a B16 mouse melanoma cell line and attenuate in vivo melanoma tumor growth. Oncol Rep. 2006;15(1):107–12. PubMed PMID: 16328041. 16328041

[20] Min KJ, Seo BR, Bae YC, Yoo YH, Kwon TK. Antipsychotic agent thioridazine sensitizes renal carcinoma Caki cells to TRAIL-induced apoptosis through reactive oxygen species-mediated inhibition of Akt signaling and downregulation of Mcl-1 and c-FLIP(L). Cell Death Dis. 2014;5(2):e1063. 10.1038/cddis.2014.35 . PubMed Central PMCID: PMC3944252. PMC3944252 24556678

[21] Cheng HW, Liang YH, Kuo YL, Chuu CP, Lin CY, Lee MH, et al. Identification of thioridazine, an antipsychotic drug, as an antiglioblastoma and anticancer stem cell agent using public gene expression data. Cell Death Dis. 2015;6(5):e1753. 10.1038/cddis.2015.77 . PubMed PMID: 25950483; PubMed Central PMCID: PMC4669717. PMC4669717 25950483

[22] Lin X, Zhang J, Wang X, Lin G, Chen T. Pre-activation with TLR7 in combination with thioridazine and loratadine promotes tumoricidal T-cell activity in colorectal cancer. Anticancer Drugs. 2020;31(10):989–96. 10. 1097/CAD.0000000000000972 PubMed PMID: 32694422. 10.1097/CAD.0000000000000972 32694422

[23] Tran TH, Kao M, Liu HS, Hong YR, Su Y, Huang CYF. Repurposing thioridazine for inducing immunogenic cell death in colorectal cancer via eIF2α/ATF4/CHOP and secretory autophagy pathways. Cell Commun Signal CCS. 2023;21(1):184. 10.1186/s12964-023-01190-5 . PubMed PMID: 37488534; PubMed Central PMCID: PMC10364410. PMC10364410 37488534

[24] Singh V, Jaiswal PK, Ghosh I, Koul HK, Yu X, De Benedetti A. Targeting the TLK1/NEK1 DDR axis with Thioridazine suppresses outgrowth of androgen independent prostate tumors. Int J Cancer. 2019;145(4):1055–67. 10.1002/ijc.32200 . PubMed PMID: 30737777; PubMed Central PMCID: PMC6617729. PMC6617729 30737777

[25] Luo Y, Yu T, Li X, Qian G. Thioridazine Enhances Cisplatin-Induced DNA Damage in Cisplatin-Resistant Human Lung Cancer Cells. Wei X, editor. Evid Based Complement Alternat Med. 2022;2022:1–8. 10.1155/2022/3702665 PMC8986366 35399625

[26] Chu CW, Ko HJ, Chou CH, Cheng TS, Cheng HW, Liang YH, et al. Thioridazine Enhances P62-Mediated Autophagy and Apoptosis Through Wnt/β-Catenin Signaling Pathway in Glioma Cells. Int J Mol Sci. 2019;20(3). 10.3390/ijms20030473 . PubMed Central PMCID: PMC6386927. PMC6386927 30678307

[27] Qian G, Dai L, Yu T. Thioridazine Sensitizes Cisplatin Against Chemoresistant Human Lung and Ovary Cancer Cells. DNA Cell Biol. 2019;38(7):718–24. 10.1089/dna.2019.4715 . PubMed PMID: 31188023. 31188023

[28] Jiang X, Chen Z, Shen G, Jiang Y, Wu L, Li X, et al. Psychotropic agent thioridazine elicits potent in vitro and in vivo anti-melanoma effects. Biomed Pharmacother. 2018;97:833–7. 10.1016/j.biopha.2017.11.012. 29136758

[29] Kang S, Dong SM, Kim BR, Park MS, Trink B, Byun HJ, et al. Thioridazine induces apoptosis by targeting the PI3K/Akt/mTOR pathway in cervical and endometrial cancer cells. Apoptosis Int J Program Cell Death. 2012;17(9):989–97. 10.1007/s10495-012-0717-2 . PubMed PMID: 22460505; PubMed Central PMCID: PMC3413814. PMC3413814 22460505

[30] Yue H, Huang D, Qin L, Zheng Z, Hua L, Wang G, et al. Targeting Lung Cancer Stem Cells with Antipsychological Drug Thioridazine. BioMed Res Int. 2016;2016:1–7. 10.1155/2016/6709828. PMC4983333 27556038

[31] Wang Y, Xia L, Lin J, Gong L, Xia Y, Xu Y, et al. Thioridazine combined with carboplatin results in synergistic inhibition of triple negative breast cancer by targeting cancer stem cells. Transl Oncol. 2022;26:101549. 10.1016/j.tranon.2022.101549 . PubMed PMID: 36191461; PubMed Central PMCID: PMC9530598. PMC9530598 36191461

[32] She X, Gao Y, Zhao Y, Yin Y, Dong Z. A high-throughput screen identifies inhibitors of lung cancer stem cells. Biomed Pharmacother. 2021;140:111748. 10.1016/j.biopha.2021.111748. 34044271

[33] Camuzard O, Trojani MC, Santucci-Darmanin S, Pagnotta S, Breuil V, Carle GF, et al. Autophagy in Osteosarcoma Cancer Stem Cells Is Critical Process which Can Be Targeted by the Antipsychotic Drug Thioridazine. Cancers. 2020;12(12):3675. 10.3390/cancers12123675 . PubMed PMID: 33297525; PubMed Central PMCID: PMC7762415. PMC7762415 33297525

[34] Shen J, Ma B, Zhang X, Sun X, Han J, Wang Y, et al. Thioridazine has potent antitumor effects on lung cancer stem-like cells. Oncol Lett. 2017;13(3):1563–8. 10.3892/ol.2017.5651. PMC5403693 28454291

[35] Shen S, Faouzi S, Souquere S, Roy S, Routier E, Libenciuc C, et al. Melanoma Persister Cells Are Tolerant to BRAF/MEK Inhibitors via ACOX1-Mediated Fatty Acid Oxidation. Cell Rep. 2020;33(8):108421. 10.1016/j.celrep.2020.108421. 33238129

[36] Matteoni S, Matarrese P, Ascione B, Ricci-Vitiani L, Pallini R, Villani V, et al. Chlorpromazine induces cytotoxic autophagy in glioblastoma cells via endoplasmic reticulum stress and unfolded protein response. J Exp Clin Cancer Res. 2021;40(1):347. 10.1186/s13046-021-02144-w. PMC8569984 34740374

[37] Park MS, Dong SM, Kim BR, Seo SH, Kang S, Lee EJ, et al. Thioridazine inhibits angiogenesis and tumor growth by targeting the VEGFR-2/PI3K/mTOR pathway in ovarian cancer xenografts. Oncotarget. 2014;5(13):4929–34. 2063 PubMed PMID: 24952635; PubMed Central PMCID: PMC4148111. 10.18632/oncotarget.2063 PMC4148111 24952635

[38] Manoharan GB, Okutachi S, Abankwa D. Potential of phenothiazines to synergistically block calmodulin and reactivate PP2A in cancer cells. PLoS ONE. 2022;17(5):e0268635. 10.1371/journal.pone.0268635 . PubMed PMID: 35617282; PubMed Central PMCID: PMC9135253. PMC9135253 35617282

[39] Mehrabi SF, Elmi S, Nylandsted J. Repurposing phenothiazines for cancer therapy: compromising membrane integrity in cancer cells. Front Oncol. 2023;13:1320621. 10.3389/fonc. 2023.1320621 PubMed PMID: 38074670; PubMed Central PMCID: PMC10701681. PMC10701681 38074670

[40] Ghinet A, Moise IM, Rigo B, Homerin G, Farce A, Dubois J, et al. Studies on phenothiazines: New microtubule-interacting compounds with phenothiazine A-ring as potent antineoplastic agents. Bioorg Med Chem. 2016;24(10):2307–17. 10.1016/j.bmc.2016.04.001 . PubMed PMID: 27073050. 27073050

[41] Rácz B, Spengler G. Repurposing Antidepressants and Phenothiazine Antipsychotics as Efflux Pump Inhibitors in Cancer and Infectious Diseases. Antibiotics. 2023;12(1):137. 10.3390/antibiotics12010137. PMC9855052 36671340

[42] Liu S, Yao S, Yang H, Liu S, Wang Y, Autophagy. Regulator of cell death. Cell Death Dis. 2023;14(10):1–17. 10.1038/s41419-023-06154-8. PMC10551038 37794028

[43] Lopes RM, Souza ACS, Otręba M, Rzepecka-Stojko A, Tersariol ILS, Rodrigues T. Targeting autophagy by antipsychotic phenothiazines: potential drug repurposing for cancer therapy. Biochem Pharmacol. 2024;222:116075. 10.1016/j.bcp.2024.116075. 38395266

[44] Zhou W, Martinez-Garcia E, Sarnow K, Kanli G, Nazarov PV, Li Y, et al. Development of a highly differentiated rat brain organoid model for exploring glioblastoma invasion dynamics and therapy. Neuro-Oncol. 2025;noaf271. 10.1093/neuonc/noaf271. PMC13070497 41284925

[45] Hanahan D, Weinberg RA. Hallmarks of cancer: the next generation. Cell. 2011;144(5):646–74. 10.1016/j.cell.2011. 02.013 PubMed PMID: 21376230. 21376230

[46] Larkin J, Chiarion-Sileni V, Gonzalez R, Grob JJ, Rutkowski P, Lao CD, et al. Five-Year Survival with Combined Nivolumab and Ipilimumab in Advanced Melanoma. N Engl J Med. 2019;381(16):1535–46. doi:10.1056/NEJMoa1910836 PubMed PMID: 31562797. 10.1056/NEJMoa1910836 31562797

[47] Amaral T, Ottaviano M, Arance A, Blank C, Chiarion-Sileni V, Donia M, et al. Cutaneous melanoma: ESMO Clinical Practice Guideline for diagnosis, treatment and follow-up☆. Ann Oncol. 2025;36(1):10–30. .006 PubMed PMID: 39550033. 10.1016/j.annonc.2024.11.006 PMC7618628 39550033

[48] Berrouet C, Dorilas N, Rejniak KA, Tuncer N. Comparison of Drug Inhibitory Effects (IC50) in Monolayer and Spheroid Cultures. Bull Math Biol. 2020;82(6):68. 10.1007/s11538-020-00746-7 . PubMed PMID: 32495209; PubMed Central PMCID: PMC9773863. PMC9773863 32495209

[49] Bjerkvig R, Laerum OD, Mella O. Glioma cell interactions with fetal rat brain aggregates in vitro and with brain tissue in vivo. Cancer Res. 1986;46(8):4071–9. PubMed PMID: 3731074. 3731074

[50] Radke J, Schumann E, Onken J, Koll R, Acker G, Bodnar B, et al. Decoding molecular programs in melanoma brain metastases. Nat Commun. 2022;13(1):7304. 10.1038/s41467-022-34899-x. PMC9701224 36435874

[51] Feitelson MA, Arzumanyan A, Kulathinal RJ, Blain SW, Holcombe RF, Mahajna J, et al. Sustained proliferation in cancer: mechanisms and novel therapeutic targets. Semin Cancer Biol. 2015;35(Suppl):S25–54. 10.1016/j.semcancer.2015.02.006 . PubMed PMID: 25892662; PubMed Central PMCID: PMC4898971. PMC4898971 25892662

[52] Dixon AJ, Sladden M, Zouboulis CC, Popescu CM, Nirenberg A, Steinman HK, et al. Primary Cutaneous Melanoma—Management in 2024. J Clin Med. 2024;13(6):6. 10.3390/jcm13061607. PMC10971470 38541831

[53] Quintana E, Shackleton M, Sabel MS, Fullen DR, Johnson TM, Morrison SJ. Efficient tumor formation by single human melanoma cells. Nature. 2008;456(7222):593–8. 10.1038/nature07567 . PubMed PMID: 19052619; PubMed Central PMCID: PMC2597380. PMC2597380 19052619

[54] Carneiro BA, El-Deiry WS. Targeting apoptosis in cancer therapy. Nat Rev Clin Oncol. 2020;17(7):395–417. 10.1038/s41571-020-. 0341-y PubMed PMID: 32203277; PubMed Central PMCID: PMC8211386. PMC8211386 32203277

[55] van Engeland M, Nieland LJW, Ramaekers FCS, Schutte B, Reutelingsperger CPM. Annexin V-Affinity assay: A review on an apoptosis detection system based on phosphatidylserine exposure. Cytometry. 1998;31(1):1–9. 10.1002/(SICI)1097-0320. ::AID-CYTO1>3.0.CO;2-R. 9450519

[56] Mauvezin C, Neufeld TP. Bafilomycin A1 disrupts autophagic flux by inhibiting both V-ATPase-dependent acidification and Ca-P60A/SERCA-dependent autophagosome-lysosome fusion. Autophagy. 2015;11(8):1437–8. 10.1080/15548627.2015.1066957 . PubMed PMID: 26156798; PubMed Central PMCID: PMC4590655. PMC4590655 26156798

[57] Ponpuak M, Mandell MA, Kimura T, Chauhan S, Cleyrat C, Deretic V. Secretory autophagy. Curr Opin Cell Biol. 2015;35:106–16. 10.1016/j.ceb. 2015.04.016 PubMed PMID: 25988755; PubMed Central PMCID: PMC4529791. PMC4529791 25988755

[58] Martinelli S, Anderzhanova EA, Bajaj T, Wiechmann S, Dethloff F, Weckmann K, et al. Stress-primed secretory autophagy promotes extracellular BDNF maturation by enhancing MMP9 secretion. Nat Commun. 2021;12(1):4643. 10.1038/s41467-021-24810-5. PMC8324795 34330919

[59] Daniel WA, Wójcikowski J. The role of lysosomes in the cellular distribution of thioridazine and potential drug interactions. Toxicol Appl Pharmacol. 1999;158(2):115–24. 10.1006/taap.1999. .8688 PubMed PMID: 10406926. 10406926

[60] Amaral L, Viveiros M, Thioridazine. A Non-Antibiotic Drug Highly Effective, in Combination with First Line Anti-Tuberculosis Drugs, against Any Form of Antibiotic Resistance of Mycobacterium tuberculosis Due to Its Multi-Mechanisms of Action. Antibiotics. 2017;6(1). 10.3390/antibiotics6010003. PMC5372983 28098814

[61] Beach SR, Celano CM, Noseworthy PA, Januzzi JL, Huffman JC. QTc prolongation, torsades de pointes, and psychotropic medications. Psychosomatics. 2013;54(1):1–13. 10.1016/j.psym.2012. 11.001 PubMed PMID: 23295003. 23295003

[62] Thioridazine - an. overview | ScienceDirect Topics [Internet]. [cited 2026 Feb 26]. Available from: https://www.sciencedirect.com/topics/agricultural-and-biological-sciences/thioridazine

[63] Hartigan-Go K, Bateman N, Nyberg G, Mårtensson E, Thomas SHL. Concentration-related pharmacodynamic effects of thioridazine and its metabolites in humans. Clin Pharmacol Ther. 1996;60(5):543–53. 10.1016/S0009-9236(96)90150-2. 8941027

[64] Thioridazine - an. overview | ScienceDirect Topics [Internet]. [cited 2026 Feb 26]. Available from: https://www.sciencedirect.com/topics/immunology-and-microbiology/thioridazine

[65] Berecz R, de la Rubia A, Dorado P, Fernández-Salguero P, Dahl ML, LLerena A. Thioridazine steady-state plasma concentrations are influenced by tobacco smoking and CYP2D6, but not by the CYP2C9 genotype. Eur J Clin Pharmacol. 2003;59(1):45–50. 10.1007/s00228-003-0576-4. 12682803

[66] Svendsen CN, Hrbek CC, Casendino M, Nichols RD, Bird ED. Concentration and distribution of thioridazine and metabolites in schizophrenic post-mortem brain tissue. Psychiatry Res. 1988;23(1):1–10. 10.1016/0165-1781(88)90029-7. 3363012

