# Supplementary material for: Repurposing thioridazine as a novel treatment for melanoma brain metastasis
Source: Cancer Cell Int. 2026 Aug 6;26:355. doi: 10.1186/s12935-026-04440-x (PMC13729370; doi:10.1186/s12935-026-04440-x)
Supplement: Supplementary file 1 [file 12935_2026_4440_MOESM1_ESM.docx]

**Supplementary Figure 1**. Raw data from monolayer CCK-8 viability assay of thioridazine in normal human astrocytes (NHA) and MBM (H1, and H2) cell lines. **A)** NHA cell line viability overview. **B)** H1 cell line viability overview. **C)** H2 cell line viability overview.

**Supplementary Figure 2**. Raw data from monolayer CCK-8 viability assay of thioridazine on MBM (H3 and H10) cell lines. **A)** H3 cell line viability overview. **B)** H10 cell line viability overview.

**Supplementary Figure 3**. Raw data from CCK-8 viability assays of thioridazine on anchorage-independent cell cultures of MBM (H1, H2, and H10) cell lines. H3 did not grow in an anchorage independent condition. Data points at 1 μM thioridazine were generally omitted from the analysis due to showing abnormal viabilities exceeding those of the untreated controls. **A)** H1 cell line viability overview. **B)** H2 cell line viability overview. **C)** H10 cell line viability overview.

**Supplementary Figure 4**. Raw data from IncuCyte analysis of the proliferative capacity of the H1 MBM cell line following thioridazine treatment over time. Shown is run 1 of the H1 cell line over 72 h following treatment with 2.5, 5, 10, or 20 μM thioridazine compared with an untreated control.

**Supplementary Figure 5**. Raw data from IncuCyte analysis of the proliferative capacity of the H1 MBM cell line following thioridazine treatment over time. Shown is run 2 of the H1 cell line over 72 h following treatment with 2.5, 5, 10, or 20 μM thioridazine compared with an untreated control.

**Supplementary Figure 6**. Raw data from IncuCyte analysis of the proliferative capacity of the H1 MBM cell line following thioridazine treatment over time. Shown is run 3 of the H1 cell line over 70 h following treatment with 2.5, 5, 10, or 20 μM thioridazine compared with an untreated control.

**Supplementary Figure 7**. Raw data from IncuCyte analysis of the proliferative capacity of the H2 MBM cell line following thioridazine treatment over time. Shown is run 1 of the H2 cell line over 72 h following treatment with 2.5, 5, 10, or 20 μM thioridazine compared with an untreated control.

**Supplementary Figure 8**. Raw data from IncuCyte analysis of the proliferative capacity of the H2 MBM cell line following thioridazine treatment over time. Shown is run 2 of the H2 cell line over 72 h following treatment with 2.5, 5, 10, or 20 μM thioridazine compared with an untreated control.

**Supplementary Figure 9**. Raw data from IncuCyte analysis of the proliferative capacity of the H2 MBM cell line following thioridazine treatment over time. Shown is run 3 of the H2 cell line over 72 h following treatment with 2.5, 5, 10, or 20 μM thioridazine compared with an untreated control.

**Supplementary Figure 10**. Raw data from IncuCyte analysis of the proliferative capacity of the H3 MBM cell line following thioridazine treatment over time. Shown is run 1 of the H3 cell line over 72 h following treatment with 2.5, 5, 10, or 20 μM thioridazine compared with an untreated control.

**Supplementary Figure 11**. Raw data from IncuCyte analysis of the proliferative capacity of the H3 MBM cell line following thioridazine treatment over time. Shown is run 2 of the H3 cell line over 72 h following treatment with 2.5, 5, 10, or 20 μM thioridazine compared with an untreated control.

**Supplementary Figure 12**. Raw data from IncuCyte analysis of the proliferative capacity of the H3 MBM cell line following thioridazine treatment over time. Shown is run 3 of the H3 cell line over 72 h following treatment with 2.5, 5, 10, or 20 μM thioridazine compared with an untreated control.

**Supplementary Figure 13**. Raw data from IncuCyte analysis of the proliferative capacity of the H10 MBM cell line following thioridazine treatment over time. Shown is run 1 of the H10 cell line over 72 h following treatment with 2.5, 5, 10, or 20 μM thioridazine compared with an untreated control.

**Supplementary Figure 14**. Raw data from IncuCyte analysis of the proliferative capacity of the H10 MBM cell line following thioridazine treatment over time. Shown is run 2 of the H10 cell line over 72 h following treatment with 2.5, 5, 10, or 20 μM thioridazine compared with an untreated control.

**Supplementary Figure 15**. Raw data from IncuCyte analysis of the proliferative capacity of the H10 MBM cell line following thioridazine treatment over time. Shown is run 3 of the H10 cell line over 72 h following treatment with 2.5, 5, 10, or 20 μM thioridazine compared with an untreated control.

**Supplementary Figure 16**. Raw data from an IncuCyte scratch-wound analysis of the migratory capacity of the H1 MBM cell line following thioridazine treatment over time. Shown is run 1 of the H1 cell line over 70 h following treatment with 2.5, 5, or 10 μM thioridazine compared with an untreated control.

**Supplementary Figure 17**. Raw data from an IncuCyte scratch-wound analysis of the migratory capacity of the H1 MBM cell line following thioridazine treatment over time. Shown is run 2 of the H1 cell line over 72 h following treatment with 2.5, 5, or 10 μM thioridazine compared with an untreated control.

**Supplementary Figure 18**. Raw data from an IncuCyte scratch-wound analysis of the migratory capacity of the H1 MBM cell line following thioridazine treatment over time. Shown is run 3 of the H1 cell line over 72 h following treatment with 2.5, 5, or 10 μM thioridazine compared with an untreated control.

**Supplementary Figure 19**. Raw data from an IncuCyte scratch-wound analysis of the migratory capacity of the H2 MBM cell line following thioridazine treatment over time. Shown is run 1 of the H2 cell line over 70 h following treatment with 2.5, 5, or 10 μM thioridazine compared with an untreated control. Note the missing datapoint at 42 h.

**Supplementary Figure 20**. Raw data from an IncuCyte scratch-wound analysis of the migratory capacity of the H2 MBM cell line following thioridazine treatment over time. Shown is run 2 of the H2 cell line over 72 h following treatment with 2.5, 5, or 10 μM thioridazine compared with an untreated control.

**Supplementary Figure 21**. Raw data from an IncuCyte scratch-wound analysis of the migratory capacity of the H2 MBM cell line following thioridazine treatment over time. Shown is run 3 of the H2 cell line over 72 h following treatment with 2.5, 5, or 10 μM thioridazine compared with an untreated control.

**Supplementary Figure 22**. Raw data from an IncuCyte scratch-wound analysis of the migratory capacity of the H3 MBM cell line following thioridazine treatment over time. Shown is run 1 of the H3 cell line over 72 h following treatment with 2.5, 5, or 10 μM thioridazine compared with an untreated control.

**Supplementary Figure 23**. Raw data from an IncuCyte scratch-wound analysis of the migratory capacity of the H3 MBM cell line following thioridazine treatment over time. Shown is run 2 of the H3 cell line over 72 h following treatment with 2.5, 5, or 10 μM thioridazine compared with an untreated control.

**Supplementary Figure 24**. Raw data from an IncuCyte scratch-wound analysis of the migratory capacity of the H3 MBM cell line following thioridazine treatment over time. Shown is run 3 of the H3 cell line over 72 h following treatment with 2.5, 5, or 10 μM thioridazine compared with an untreated control.

**Supplementary Figure 25**. Raw data from an IncuCyte scratch-wound analysis of the migratory capacity of the H10 MBM cell line following thioridazine treatment over time. Shown is run 1 of the H10 cell line over 72 h following treatment with 2.5, 5, or 10 μM thioridazine compared with an untreated control.

**Supplementary Figure 26**. Raw data from an IncuCyte scratch-wound analysis of the migratory capacity of the H10 MBM cell line following thioridazine treatment over time. Shown is run 2 of the H10 cell line over 72 h following treatment with 2.5, 5, or 10 μM thioridazine compared with an untreated control.

**Supplementary Figure 27**. Raw data from an IncuCyte scratch-wound analysis of the migratory capacity of the H10 MBM cell line following thioridazine treatment over time. Shown is run 3 of the H10 cell line over 72 h following treatment with 2.5, 5, or 10 μM thioridazine compared with an untreated control.

**Supplementary Figure 28**. Raw data from colony forming assays of the H1 and H2 MBM cell lines following treatment with 2.5, 5, or 10 μM thioridazine compared with an untreated control. H3 and H10 did not form sufficient colonies for analysis.

**Supplementary Figure 29.** Flow cytometric analysis of apoptosis following thioridazine treatment. (**a**) Representative dot plots showing the response of the H1 and H2 MBM cell lines treated with 10 µM thioridazine for 24 h compared with untreated cells. Annexin V labels apoptotic cells, while propidium iodide (PI) labels necrotic cells. Quadrant interpretation: Lower left (Q4) – viable cells, lower right (Q3) – early apoptotic cells, upper right (Q2) – late apoptotic cells, upper left (Q1) – necrotic or late apoptotic cells.

**Supplementary Figure 30**. Example of the gating strategy performed for flow cytometric analysis in FlowJo.

**Supplementary Figure 31.** Full flow cytometry batch reports for the H1, H2, H3, and H10 cell lines treated with thioridazine at 2.5, 5, 10, or 20 µM for 72 h compared with untreated controls.

**Supplementary Figure 32**. Raw data from flow cytometric analysis of apoptosis in the H1, H2, H3, and H10 cell lines following treatment with 10 μM thioridazine compared with untreated controls.

**Supplementary Figure 33.** Full GAPDH blots used as loading controls for normalization of apoptosis-related protein band intensities (N = 3).

**Supplementary Figure 34.** Cleaved caspase-3 blot overview. (**a**) Side-by-side comparison of all cleaved caspase-3 Western blot membranes with their corresponding GAPDH loading controls. (**b**) Original full blot images of the membranes used to assess cleaved caspase-3 expression by chemiluminescence (N = 3).

**Supplementary Figure 35.** PARP1 blot overview. (**a**) Side-by-side comparison of all PARP1 Western blot membranes with their corresponding GAPDH loading controls. (**b**) Original full blot images of the membranes used to assess PARP1 expression by chemiluminescence (N = 3).

**Supplementary Figure 36.** Bcl-2 blot overview. (**a**) Side-by-side comparison of all Bcl-2 Western blot membranes with their corresponding GAPDH loading controls. (**b**) Original full blot images of the membranes used to assess Bcl-2 expression by chemiluminescence (N = 3).

**Supplementary Figure 37.** Full GAPDH blots used as loading controls for normalization of autophagy-related protein band intensities (N = 3).

**Supplementary Figure 38.** p62 blot overview. (**a**) Side-by-side comparison of all p62 Western blot membranes with their corresponding GAPDH loading controls. (**b**) Original full blot images of the membranes used to assess p62 expression by chemiluminescence (N = 3).

 **Supplementary Figure 39.** Western blots of baseline LC3A/B expression in our MBM cell lines. Blots showing LC3A/B expression following 72 h incubation with 7.5 µM thioridazine as compared to untreated controls in our MBM cell lines (H1, H2, H3, and H10).

**Supplementary Figure 40.** Western blots showing baseline caspase-3 in our MBM cell lines. (**a**) A representative Western blot showing baseline caspase-3 expression in the H1, H2, H3, and H10 MBM cell lines. Cells were treated with 7.5 µM thioridazine for 72 h or left untreated as controls. GAPDH was used as the loading control. (**b**) Graphical representation of a Fiji-based densitometric quantification of our caspase-3 Western blots showing fold change. Statistical analyses were performed using ordinary two-way ANOVA, ns = *P* > 0.05, *** = *P* < 0.001, N = 2 independent experiments.

**Supplementary Figure 41.** Caspase-3 full blot overview. Side-by-side comparison of all caspase-3 Western blot membranes with their corresponding GAPDH loading controls (N = 2).

**Supplementary Figure 42.** Autophagic flux experiments using bafilomycin A1 co-treatment. (**a**) Western blot showing p62 and LC3 expression in the H1 MBM cell line following treatment with 100 nM bafilomycin A1 for 4 or 12 h compared with an untreated control. (**b**) Western blot showing p62 and LC3 expression following treatment with 5 or 7.5 µM thioridazine for 72 h. Cells then either received 100 nM bafilomycin A1 (+) for 4 h or remained treated with thioridazine alone (-). These groups were compared with untreated controls (-) and cells treated with 100 nM bafilomycin A1 alone (+) for 4 h. (**c**) Graphical representation of a Fiji-based densitometric quantification of our autophagic flux experiments showing the fold change in p62 and LC3 in our H1 cell lines according to drug exposure as described above. Statistical analyses were performed using ordinary two-way ANOVA, ns = *P* > 0.05, * = *P* < 0.05, ** = *P* < 0.01, *** = *P* < 0.001, N = 3 independent experiments.

**Supplementary Figure 43.** Autophagic flux experiments: full blot overview. (**a**) Full blots showing p62 and LC3 expression in the H1 cell line following treatment with 100 nM bafilomycin A1 for 4 or 12 h compared with untreated controls. The corresponding GAPDH loading control is shown (N = 1). (**b**) Full blots showing p62 and LC3 expression in the H1 cell line following co-treatment with thioridazine and bafilomycin A1. The six lanes between the molecular weight markers correspond to the samples analyzed in Supplementary Figure 42. The rightmost lanes contain buffer samples included to maintain gel structure during electrophoresis.
